# Increasing Costs Due to Ocean Acidification Drives Phytoplankton to Be More Heavily Calcified: Optimal Growth Strategy of Coccolithophores

**DOI:** 10.1371/journal.pone.0013436

**Published:** 2010-10-15

**Authors:** Takahiro Irie, Kazuhiro Bessho, Helen S. Findlay, Piero Calosi

**Affiliations:** 1 Institute for Biodiversity and Ecosystem Dynamics, University of Amsterdam, Amsterdam, The Netherlands; 2 Department of Biology, Faculty of Sciences, Kyushu University, Fukuoka, Japan; 3 Plymouth Marine Laboratory, Plymouth, United Kingdom; 4 Marine Biology and Ecology Research Centre, University of Plymouth, Plymouth, United Kingdom; Mt. Alison University, Canada

## Abstract

Ocean acidification is potentially one of the greatest threats to marine ecosystems and global carbon cycling. Amongst calcifying organisms, coccolithophores have received special attention because their calcite precipitation plays a significant role in alkalinity flux to the deep ocean (i.e., inorganic carbon pump). Currently, empirical effort is devoted to evaluating the plastic responses to acidification, but evolutionary considerations are missing from this approach. We thus constructed an optimality model to evaluate the evolutionary response of coccolithophorid life history, assuming that their exoskeleton (coccolith) serves to reduce the instantaneous mortality rates. Our model predicted that natural selection favors constructing more heavily calcified exoskeleton in response to increased acidification-driven costs. This counter-intuitive response occurs because the fitness benefit of choosing a better-defended, slower growth strategy in more acidic conditions, outweighs that of accelerating the cell cycle, as this occurs by producing less calcified exoskeleton. Contrary to the widely held belief, the evolutionarily optimized population can precipitate larger amounts of CaCO_3_ during the bloom in more acidified seawater, depending on parameter values. These findings suggest that ocean acidification may enhance the calcification rates of marine organisms as an adaptive response, possibly accompanied by higher carbon fixation ability. Our theory also provides a compelling explanation for the multispecific fossil time-series record from ∼200 years ago to present, in which mean coccolith size has increased along with rising atmospheric CO_2_ concentration.

## Introduction

Scientists predict that increasing atmospheric CO_2_ partial pressure (*p*CO_2_), elevated by anthropogenic emissions of CO_2_, causes an increase in aqueous CO_2_ [CO_2_(aq)] and hydrogen ion concentrations [H^+^] in seawater, and a decrease in carbonate ion concentration [CO_3_^2−^] (this effect has been termed ocean acidification [1]). The carbonate undersaturation expected to arise from continued ocean acidification is considered to reduce precipitation of calcium carbonate in marine organisms that build calcareous exoskeletons [2]. In fact, laboratory experiments demonstrate that acidified seawater has a deleterious effect on the physiology in various taxa of calcifying organisms [3]–[6] including coccolithophores [7]–[11], foraminiferans [12], [13], corals [14]–[16], mollusks [17]–[19], and echinoderms [20]–[22]. In addition, dissolution of calcareous exoskeleton is confirmed by field observations in areas where volcanic or biogenic emissions have locally acidified the seawater [23], [24].

Numerous scientists expect that ocean acidification will cause shifts in extant marine ecosystems and could result in substantial biodiversity loss. Local or global extinction of calcifying primary producers would have serious impacts on their herbivores with subsequent consequences further across food webs. However over longer timescales, species have the potential to overcome these impacts by adjusting their phenotypic values through the changes in gene frequencies in populations. On shorter timescales, negative effects on higher-level ecological processes are possibly buffered by phenotypic plasticity [25], which has evolved as (pre)adaptation to existing spatial and/or temporal heterogeneities of environmental conditions. If this adaptation is possible, the effect of ocean acidification should first appear in morphological/life-history traits prior to the local extinctions and distribution change in the focal species.

Coccolithophores (Haptophyta; Prymnesiophyceae) are unicellular, marine autotrophic algae, characterized by calcitic exoskeletons (coccoliths) formed around the cell (coccosphere). Because of their worldwide abundance, coccolithophores are considered to be one of the most important producers of calcite, and play a pivotal role in the global biogeochemical cycles in terms of their ability to fix carbon into both organic and inorganic products [26]. The biogenic calcium carbonate (CaCO_3_) sequestered to deeper waters accumulates in sediments or dissolves in the deep, undersaturated waters. Both of these processes contribute to the removal of inorganic carbon from surface layers, which influences the carbon exchange between the atmosphere and the ocean. In particular, the formation of calcareous exoskeletons reduces alkalinity in surface waters and its dissolution below the thermocline supplies alkalinity to the deep waters (i.e. alkalinity flux).

Phenotypically plastic responses to acidified conditions are repeatedly evaluated using a cosmopolitan pelagic coccolithophorid species, *Emiliania huxleyi* (Lohmann). Scientists originally predicted, and then experimentally confirmed, that *E. huxleyi* had a reduced calcification rate under elevated *p*CO_2_ [8]–[12]. However, a recent study demonstrated that a different strain of *E. huxleyi* exhibited contradictory results of increased calcification rate and net primary production under high *p*CO_2_ conditions [27]. This work was subsequently followed by laboratory experiments investigating different *E. huxleyi* strain responses to ocean acidification [28], which illustrated that some strains appear to have decreased calcification rates, while other increased [29].

One possible explanation for the various plastic responses observed in *E. huxleyi* [29] and other coccolithophorid species [30] is that the reaction norms have high levels of genetic variance sufficient for adaptation to ocean acidification [31]. A strong tool to theoretically predict the adaptive response of phenotypes to acidified conditions is analyzing optimality models established in life-history theory. Historically, evolutionary ecologists interested in life history evolution have considered that natural selection favors such life history phenotypes that maximize the fitness of individuals sharing a genotype. Therefore, models are analyzed in the mathematical optimization framework for calculating the evolutionary equilibrium state(s) of optimal life history that falls within a particular phenotypic range determined by specific constraints [32]–[34]. Assessing an optimal life history model where a calcareous exoskeleton of marine organisms is considered to be a defensive organ [35], [36] should provide us with a significant insight into the life history evolution of these organisms under conditions of ocean acidification.

In the present study, we model a growth schedule of coccolith-bearing coccolithophores in the asexual reproduction phase, aiming to theoretically evaluate how natural selection alters their optimal growth strategy as ocean acidification progresses. Ongoing global climate change potentially causes multiple changes in coccolithophore habitats, including temperature, current, nutrient availability, oxygen supply, and grazing risk. Yet we specifically focus on the marginal effects due to ocean acidification, because our purpose is to provide a theoretically well-grounded working hypothesis which can be tested against empirical hypotheses rather than predict the future. In particular, our interest is in how acidification-driven costs affect the behavior of optimal sizes of the coccosphere and coccoliths as well as the optimal generation time, assuming that an acidified environment enhances photosynthesis, leads to a higher physiological maintenance cost, imposes more energy to form calcareous exoskeleton, accelerates dissolution of exoskeleton, and/or inflates a defensible mortality risk. We also evaluated the acidification impact on the total amount of precipitated CaCO_3_ during the bloom, which has a greater importance in a geochemical context.

## Methods

We provide a theoretical model on the growth schedule of coccolith-bearing coccolithophores in the asexual reproduction phase, aiming to predict how natural selection alters their phenotypes as ocean acidification progresses and to evaluate the resultant change in carbon fixation ability. All model variables and parameters are listed in Table 1. In the following model, we have kept the functional form to be as general as possible because accurate quantitative relationships among the physiological and ecological variables are still largely unknown in coccolithophores (see Discussion). Accordingly, the subsequent mathematical analysis was concentrated on the qualitative behavior of the system rather than quantitative prediction of particular variables.

**Table 1:** The variables and parameters in the model.

Symbol	Interpretation
*a*	Net production coefficient
*s*	Cost of calcification
α	Dissolution factor
β	Dissolution exponent
*P*	Defensible mortality risk
*k*	Metabolic exponent
*a*_p_	Photosynthetic coefficient
*a*_m_	Maintenance coefficient
*k*_p_	Photosynthetic exponent
*k*_m_	Maintenance exponent
*t*	Cell ontogenetic time elapsed from the last binary fission
*T*	Time interval between two successive binary fission ( = generation time)
*u*(*t*)	Fractional energy allocation to coccolith production
*V*(*t*)	Coccosphere volume
*C*(*t*)	Total coccolith volume
*L*(*t*)	Probability of surviving from the last binary fission until time *t*
δ	Coccolith to coccosphere volume ratio
*g*(*t*)	Instantaneous mortality rate
*q*	Defense efficiency exponent
*r*	Intrinsic rate of population increase
τ	Time elapsed from the onset of bloom
*W*(τ)	Accumulated total amount of precipitated CaCO_3_
*W*_1_(τ)	Total weight of coccoliths produced by the individuals that died during the bloom
*W*_2_(τ)	Total weight of coccoliths produced by the individuals that survived until the end of bloom
*N*_0_	Population size at the onset of bloom
*N_D_*(τ)	Total number of daughter cells that emerge during the bloom
Ξ	Expected amount of coccolith left by a daughter cell that dies for a generation

### Life cycle

As with the other eukaryotes, life cycles of coccolithophores consist of haploid and diploid phases, between which the alternations of generations are accompanied by syngamy (haploid to diploid) or meiosis (diploid to haploid). *Emiliania huxleyi* bears coccoliths only during diploid phase. The primary mode of reproduction is asexual binary fission, which repeats within the same ploidy level via mitosis [37]. Our model considers coccolith-bearing individuals multiplying by vegetative binary fission, in which a generation starts at the ontogenetic time, *t* = 0, and ends at *t* = *T* with the next binary fission.

### Acidification-sensitive energetic costs

Coccolithophorids have chloroplasts to capture light energy by photosynthetic pigments contained therein. We assumed that the energy acquired by photosynthesis is positively related to coccosphere volume, *V*, through a power function, *a*_p_ *V^k^* ^p^, where *a*_p_ (>0) is the photosynthetic coefficient and *k*_p_ (>0) is the photosynthetic exponent. The latter is likely to be less than 1 because the density of photosynthetic pigments is expected to decrease as the phytoplankton cell volume increases [38]. For simplicity, we consider that photosynthetic rate is independent of exoskeletal size, although there are untested hypotheses that coccoliths may serve as a lens to gather light [39] or operate to protect a cell from too strong light [40], [41].

The energy required by a process to maintain vital activity ( = maintenance cost) is often expressed by a power function of cell size [42]: *a*_m_ *V^k^* ^m^, where *a*_m_ (>0) is the maintenance coefficient and *k*_m_ (>0) is the maintenance exponent. The difference between these two quantities, *a*_p_ *V^k^* ^p^−*a*_m_ *V^k^* ^m^, is the energy spent on growth ( = net production). This von Bertalanffy-type assumption hinders analytically tractable optimization if *k*_p_≠*k*_m_ [43]; otherwise, the growth equation can be simplified to a power function [44]. We thus confined analysis to the case where *k*_p_ = *k*_m_ (≡*k*), in which net photosynthetic production can be simply rewritten as *aV^k^*, with *a*≡*a*_p_−*a*_m_ (>0).

Ocean acidification may positively or negatively act on the net production coefficient, *a*. A positive effect on *a* may arise by increasing the photosynthetic coefficient, *a*_p_, if photosynthesis accelerates with rising aqueous CO_2_ concentration due to ocean acidification. On the other hand, acidified seawater is considered to elevate the energy for maintaining intracellular hydrogen ion concentration through transmembrane active transport [22], [45], [46], resulting in a higher *a*_m_ value.

There should also be an energetic cost, termed the cost of calcification, to precipitate CaCO_3_ from bicarbonate and calcium ions in the coccolith vesicle. This cost, designated by *s*, includes the energy required for synthesizing calcification-related enzymes (e.g., carbonic anhydrase) as well as for producing a coccolith polysaccharide coating which surrounds the calcitic crystal [47]. The decrease in CaCO_3_ saturation state of seawater may inhibit coccolith growth, which is also described by increasing calcification cost (*s*) in our model.

### Energy allocation between state variables

**Figure 1:**
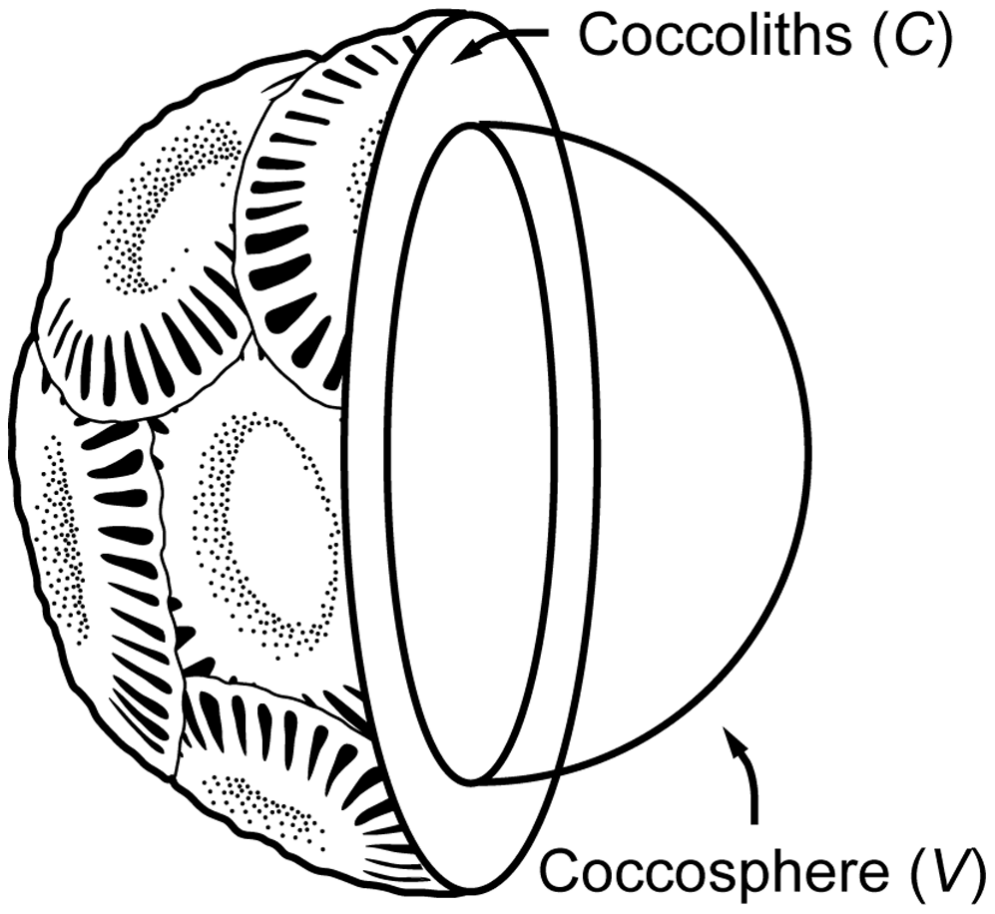
Partial cross section of a coccolithophore with coccolith layer.

The dynamic optimization procedure in optimal control theory provides the optimal time courses for “state variables”, or the size of the subsystems among which finite resources are allocated, by finding the optimal time path for a “control variable”, or resource allocation rate [48]. According to this terminology, we set both coccosphere volume, *V*, and coccolith volume, *C*, as state variables (Fig. 1).

**Figure 2:**
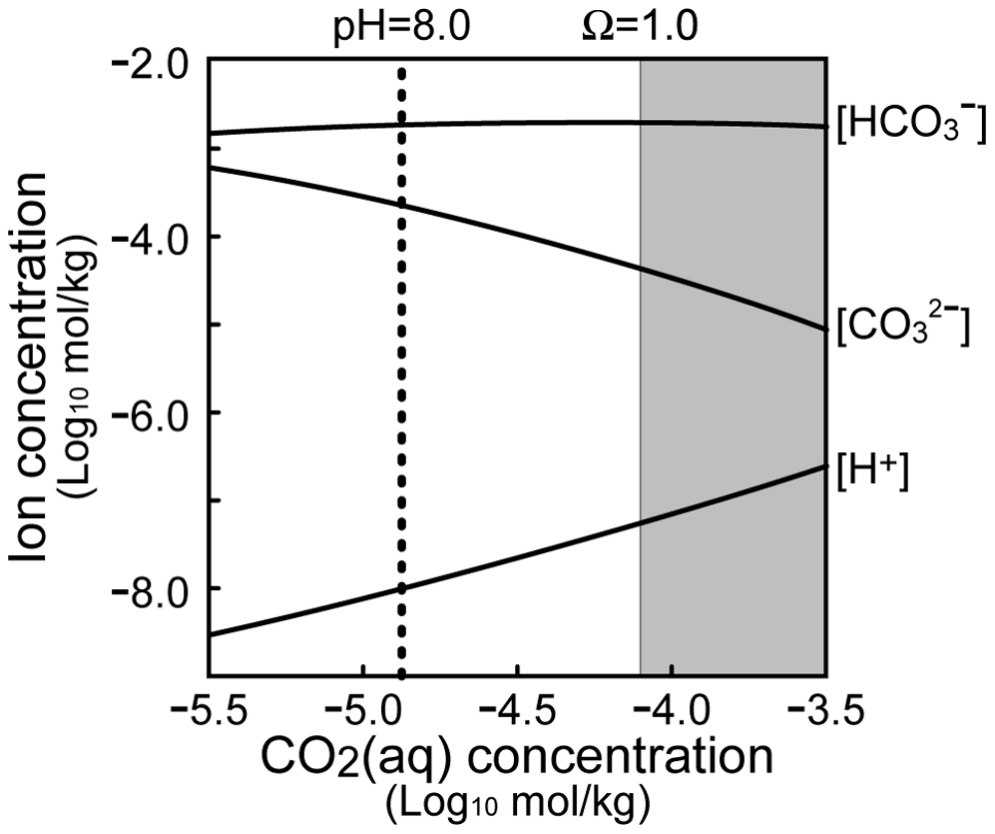
Carbonate system in seawater. Equilibrial concentrations of HCO^3−^, CO_3_^2−^, and H^+^ in response to increasing aqueous CO_2_ concentration. Parameter values are set at salinity *S* = 35, temperature *T* = 25°C, and pressure *P* = 1 atm according to Zeebe and Wolf-Gladrow [49]: total dissolved inorganic carbon (DIC) = 2.1 mmol/kg, stoichiometric equilibrium constants *K*_1_* = [HCO^3−^][H^+^]/[CO_2_] = 10^−5.86^ and *K*_2_* = [CO_3_^2−^][H^+^]/[HCO^3−^] = 10^−8.92^. The CaCO_3_ saturation state of seawater, Ω, is lower than 1 in the shaded region, in which CO_3_^2−^ concentration in seawater is assumed to be 41.6 µmol/kg (see [49]).

Assuming that net photosynthate is allocated between coccosphere growth and coccolith production, ontogenetic dynamics of the two state variables are given as simultaneous differential equations with a control variable *u*(*t*) (0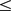 *u*<1):
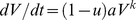
(1)

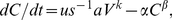
(2)where the term α*C* ^β^ indicates the dissolution of coccoliths, of which the rate depends on the values of dissolution factor, α, and dissolution exponent, β (≥0). The dissolution factor should take a positive value in CaCO_3_-undersaturated seawater; i.e., this type of cost must be particularly significant after CaCO_3_ saturation state (Ω) falls below 1 (see Fig. 2 and [49]). On the other hand, the dissolution exponent is assumed to be independent of CaCO_3_ saturation state, but may increase with the surface area to volume ratio of the exoskeleton and thus more intricate coccolith ornamentation leads to a higher β-value. Although the functional significance of coccolith is unknown, *E. huxleyi* produces more coccoliths than required to surround a coccosphere, and discards the surplus coccoliths into the surrounding seawater [50]. Note that the second term of the right-hand side in equation (2) can be also interpreted as the coccolith detachment instead of dissolution.

A coccolithophore redistributes approximately half coccoliths to a daughter cell at binary fission [50], [51]. Thus, the initial values of the state variables equate to *V*(0) = *V*(*T*)/2 and *C*(0) = *C*(*T*)/2. Furthermore, we assume that the coccolith to coccosphere volume ratio (*C*/*V* ratio) remains unchanged throughout life:
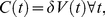
(3)where 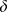
(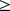
0) is the proportion coefficient. This assumption considerably facilitates solving the simultaneous differential equations (1) and (2).

### Survival probability and mortality rates

The third state variable, *L*, is the probability of survival from birth to age *t*:
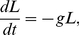
(4)and
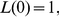
(5)where *g* is the mortality rate generally defined as a function of *V*(*t*) and *C*(*t*), or *g*(*V*(*t*),*C*(*t*)).

The behavior of our model uniquely depends on the mortality function, *g*, although the shape of this function in nature is unknown. However, there seems little doubt that the coccoliths serve as defensive organs, although producing coccoliths may also, coincidentally, have possible energetic advantage due to the biochemical linkage between calcification and photosynthesis (see Discussion). Young [39] argues that having coccoliths may protect the cell against predation, harmful short-wavelength light, osmotic, chemical, and physical shocks, and/or prevent the cell from sinking to an undesired depth through flotation regulation. In any case, coccoliths can be regarded as a defensive organ in the sense of reducing mortality risks. Therefore, we consider the case that the instantaneous mortality rate depends on coccolith size but not on coccosphere size (i.e., 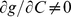 and 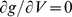). The conceivably simplest function is:
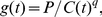
(6)where *P* and *q* are positive constants. The numerator, *P*, can be interpreted as an acidification-driven physiological cost, if having thicker coccoliths reduces its negative impact on survival. Alternatively, *P* can be also interpreted as grazing pressure.

### Coccolithophore fitness

**Figure 3:**
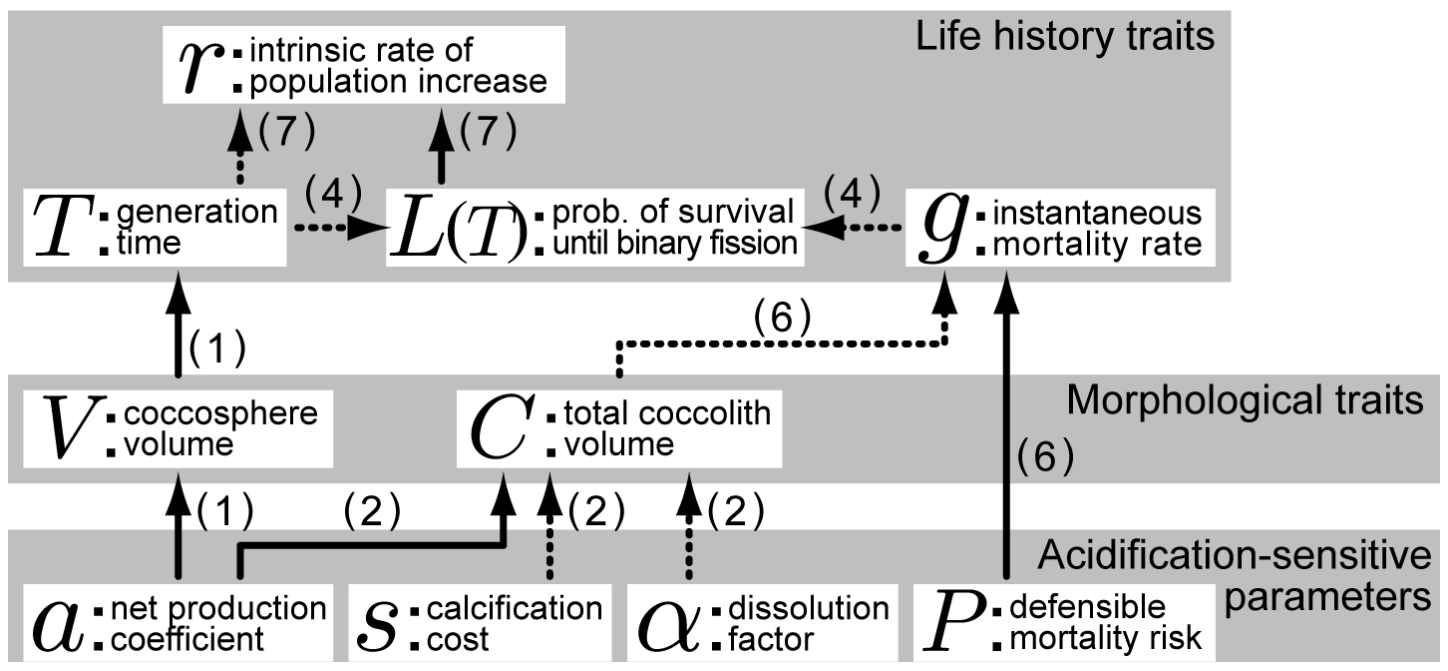
Relationships between the acidification-sensitive parameters, morphological variables, and life history variables. Arrows with solid lines indicate positive relationships and arrows with dotted lines indicate negative ones. Numbers in parentheses correspond to the equation numbers in the main text.

Since field observations suggest that bloom-forming coccolithophores (e.g., *E. huxleyi*) are *r*-strategists [52], we analyzed how the most likely changes in the acidification-sensitive parameters (i.e., *a*, *s*, α, *P*) affect the optimal life history strategy that maximizes the intrinsic rate of population increase, *r*, given as:
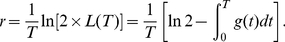
(7)The relationships between the fitness (*r*) and other parameters/variables defined above are illustrated in Fig. 3. The framework of optimization is described in Appendix S1.

### The calcification to photosynthesis ratio

Whether coccolithophorid blooms act as a source or sink of CO_2_ depends on the ratio between calcification and photosynthesis ( = C/P ratio) because the former generates CO_2_ and the latter consumes CO_2_ [50]. As shown in equations (1) and (2), net CO_2_ uptake by photosynthesis equals *aV^k^* and calcification rate is given as *u*s^−1^ *aV^k^*. Thus, the C/P ratio of an individual is *u*/*s*, if the fractional energy allocation to coccolith production (*u*) keeps constant throughout ontogeny. Otherwise, the expected C/P ratio weighted by the probability of survival until age *t* is expressed as:
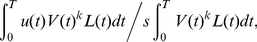
(8)because the weighted mean photosynthetic rate is
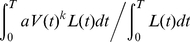
(9)and the counterpart to calcification is
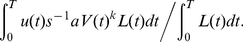
(10)


### Total amount of precipitated CaCO_3_

We also calculated the accumulated total amount of precipitated calcium carbonate, *W*, as a function of time elapsed from the onset of the bloom, τ:
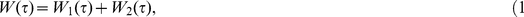
(11)where *W*_1_ is the coccoliths belonging to coccolithophores which died during the bloom and *W*_2_ is the coccoliths belonging to the individuals that survived until the end of the bloom, respectively. Denoting the population size at the onset of bloom by *N*_0_, the number of individuals at the *k*-th generation is *N*_0_(2*L*(*T*))*^k^* and the total number of daughter cells that emerge for *n* generations, *N*_D_, is
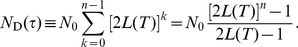
(12)Considering that the bloom continues for the period of τ (≫*T*), the amount of coccoliths produced by the individuals died during the bloom, *W*_1_(τ), equals the product of this quantity and Ξ, defined as the expected amount of coccolith left by a daughter cell that dies for a generation:
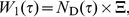
(13)where
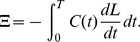
(14)On the other hand, the amount of coccoliths held by the individuals surviving until the end of the bloom is rather more simple:
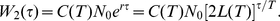
(15)


## Results

Given that exoskeletal dissolution occurs (i.e. α>0; equivalent to Ω<1 in relation to ocean acidification), the analytical tractability of this model largely depends on whether the metabolic exponent is equal to the dissolution exponent (i.e., *k* = β; see Appendix S2) or not (*k*≠β; see Appendix S3). In the former case, a large part of model's behavior can be analyzed without computer-intensive numerical approaches. The analytical results assuming *k* = β can be directly applied to the case where seawater is oversaturated in CaCO_3_ (i.e., α = 0, Ω>1), because “α = 0” is a special case of the model assuming *k* = β. Thus, we first look at the case with *k* = β in the first step (see *Analytical results*), and then we relax this restriction (see *Numerical results*).

### Defense efficiency exponent (*q*)

**Figure 4:**
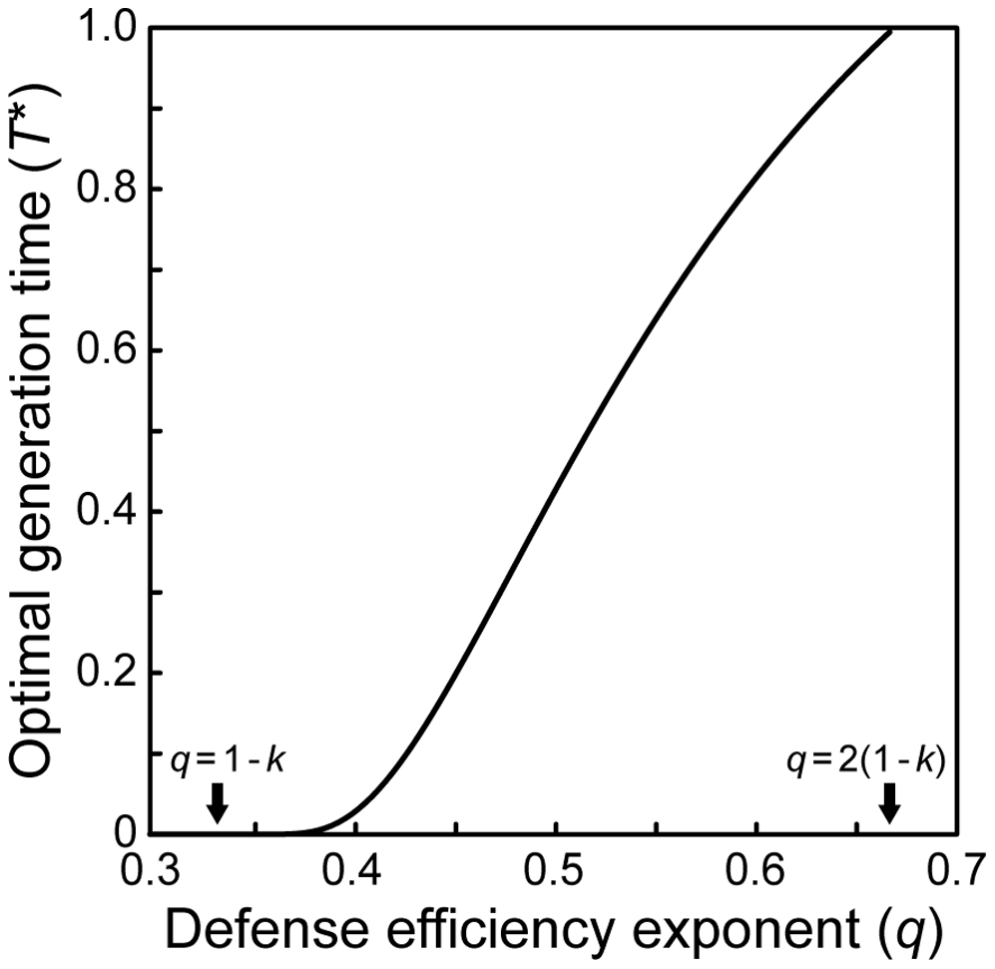
Dependence of optimal generation time on defense efficiency exponent (*q*). Based on the optimal proportion coefficient (δ*) calculated by numerically solving αδ^β^+*ak*δ−*a*(1−*k*)/*s* = 0 (see Appendix S2 equation [B3-2]), univariate static optimization was conducted by numerically choosing *T* that maximizes 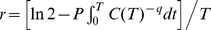 at different *q*-values (i.e., 1−*k*≤*q*≤2(1−*k*)). Parameter values: *a* = 1.0, *s* = 1.0, α = 0.0001, *P* = 0.2, *k* = β = 2/3.

The value of *q*, the exponent that determines how effective coccoliths are at reducing mortality rates, is significant in two regards: (1) the behavior of optimal life history critically depends on its value; and (2) the model's analytical tractability is ensured only when *q* has particular values. Qualitatively speaking, a relatively large value of *q* is required for the optimal growth schedule to be nontrivial; otherwise, natural selection always favors the generation time with asymptotically zero (if *q* is smaller than a certain value, 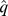; see Appendix S2 Sections 4 and 5). This pattern is reasonable because a higher fitness should be achieved by maximizing the frequency of cell division in exchange for a high mortality risk rather than spending a long time on calcification, when defense by having coccoliths is not sufficiently effective. Our analysis suggests that the threshold, 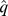, exists in the open interval 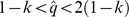, which was supported by numerical computation (Fig. 4). In the remaining sections we move on the analysis by setting *q* = 2 (1−*k*), because it is the simplest assumption leading to a non-zero generation time which enables us to obtain analytical solutions for optimal life history. Note that the quantitative relationship between *q* and *k* has little biological meaning and are therefore fixed throughout the analysis on the premise that both *q* and *k* are insensitive to the environmental change due to ocean acidification.

### Analytical results

In the case that *k* = β and *q* = 2 (1−*k*), it is analytically demonstrated that the probability of survival until binary fission (*L*(T*)) is independent of any environmental factors (i.e., *a*, *s*, α, *P*) when growth strategy is optimal (Table 2; Appendix S2 Section 5). The decrease in the net production rate (*a*) and/or the increases in calcification cost, dissolution factor, and defensible mortality risk (*s*, α, and *P*) can be regarded as ‘acidification-driven costs’, because they definitely lead to a decrease in the maximal fitness (r*), or the intrinsic rate of population increase achieved by the optimal growth schedule. These acidification-driven costs always extend optimal generation time (T*), which is underpinned by the fact that the maximized fitness (r*) is inversely proportional to the optimal generation time (T*) (Table 2; Appendix S2 Section 8). Our analysis also demonstrated that elevated calcification cost (*s*) leads to larger coccosphere and coccolith sizes (*V*(*T*)* and *C*(*T*)*) regardless of whether α = 0 or not (i.e., regardless of whether CaCO_3_ is under- or over-saturated). This is also the case for the decrease in net production coefficient (*a*) and the increases in defensible mortality risk (*P*) and dissolution factor (α). These analytical results suggest that natural selection favors a slower-growing coccolithophore with a larger cell size and more exoskeleton when suffering from a higher acidification-driven cost. These life history shifts due to ocean acidification are not mediated by any change in the optimal energy allocation (u*), which is time-invariant when *k* = β. The optimal proportion coefficient (δ* = *C*(*T*)*/*V*(*T*)*) is positively dependent on *a*, negatively on *s* and α, and independent of *P* (Table 2).

**Table 2:** Optimal phenotypic responses to ocean acidification (*k* = β).

Acidification-sensitive parameters	T*	δ*	u*	*V*(*T*)*	*C*(*T*)*	*L*(*T*)*	r*
Net production coefficient (*a*)	↓	↑	0	↓	↓	0	↑
Cost of calcification (*s*)	↑	↓	0	↑	↑	0	↓
Dissolution factor (α)	↑	↓	0	↑	↑	0	↓
Defensible mortality risk (*P*)	↑	0	0	↑	↑	0	↓

Responses of optimal life history to the increase in acidification-sensitive parameters when *k* = β, *g*(*t*) = *P*/*C*(*t*)*^q^*, and *q* = 2 (1−*k*). Zero denotes no change, ↑ an increase in the optimal value, and ↓ a decrease. Abbreviations: *T*, generation time; δ, proportion coefficient; *u*, resource allocation rate; *V*(*T*), coccosphere volume; *C*(*T*), coccolith volume; *L*(*T*), survival probability to binary fission; *r*, intrinsic rate of population increase. Asterisks indicate optimized variables.

### Numerical results

Considering that the dissolution rate of coccoliths is directly proportional to the surface area, the dissolution exponent, β, is likely to be largely dependent on the allometric relationship between the overall exoskeletal mass and the total surface area of coccoliths surrounding a coccosphere. Since the surface area of isomorphic objects with different sizes is proportional to the two-thirds power of the volume, β should be two-thirds if the total coccolith volume, *C*, changes by resizing each coccoliths in a spatially homothetic manner. Hence the dissolution exponent must take a value close to the metabolic exponent in this case (i.e. β = 2/3≈*k*). Otherwise, the dissolution exponent might be higher if, for example, the total coccolith volume entirely depends on the number of isometric coccoliths with the same shape. In this section, we will analyze the model assuming the latter case, in which β is larger than *k*.

**Figure 5:**
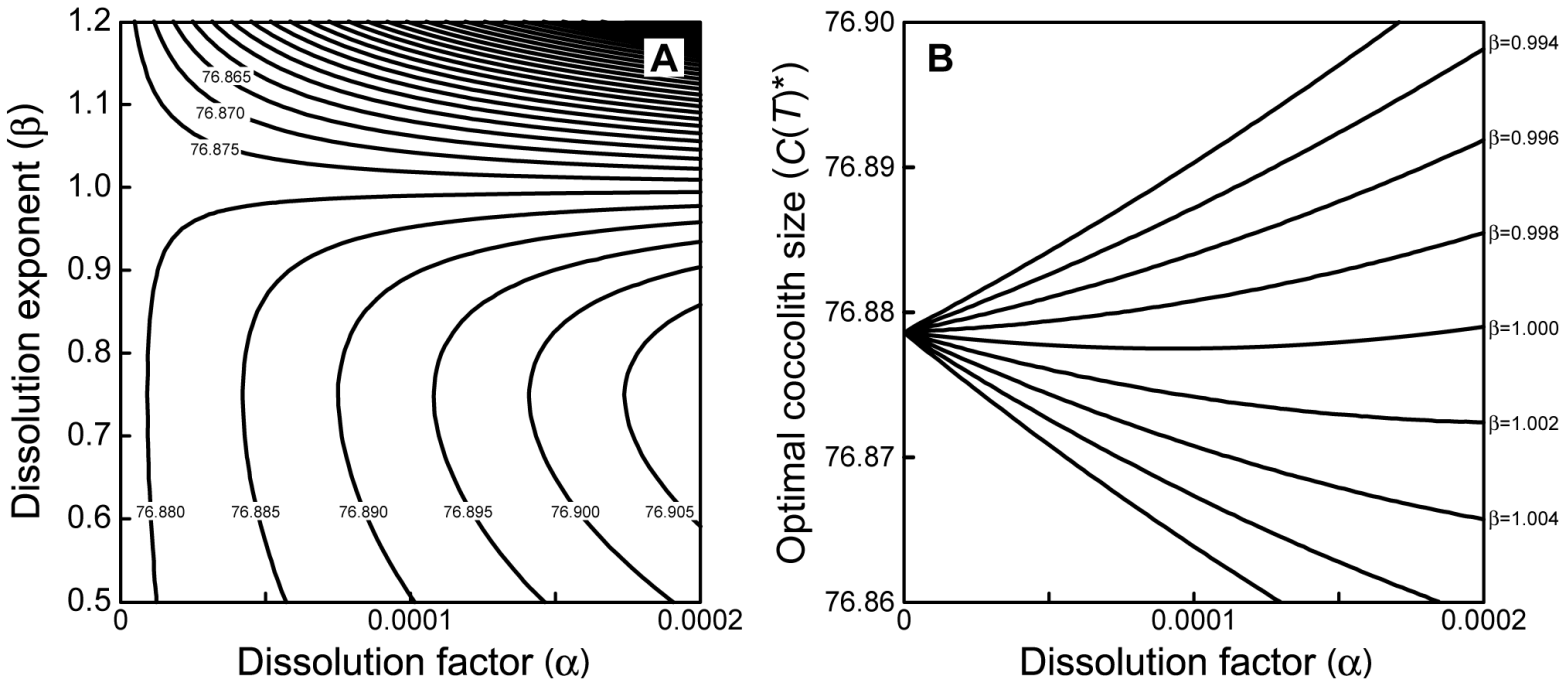
Optimal coccolith size as a function of dissolution factor (α) and dissolution exponent (β). (A) A contour plot of *C*(*T*)* in a wider range of β. (B) Detailed relationships between α and *C*(*T*)* with βs close to 1. Common parameters: *a* = 0.1, *s* = 0.001, *P* = 1.0, *k* = *q* = 2/3.

Once the assumption that *k* = β is removed, our model considerably loses the analytical tractability even assigning convenient, specific values to these exponents. To begin with, we plotted the optimal coccolith size at binary fission as a function of dissolution factor (α) and exponent (β) with the other parameters fixed (Fig. 5). Interestingly, the dependency of *C*(*T*)* on α qualitatively differs depending on β-value. Assigning two-thirds to *k*, *C*(*T*)* monotonically increases with α when β≪1 (e.g., β≈*k*), but its function shifts to a convex shape if β is close to 1; with β≫1, *C*(*T*)* shows a simple positive dependence on α (see Fig. 5B). These findings suggest that the behavior of optimal solutions may dramatically change depending on β-value, and motivate us to investigate the model's behavior in three separate cases: β≪1, β≈1, and β≫1. Since the analysis with *k* = β in the last section (*Anatical Results*) represents the case in which β≪1, here we address the other two cases by assuming β = 1 and β = 4/3, respectively, and with *k* = 2/3 in both cases (see Appendix S4 and S5). In the first step, a quick sketch of the optimal values was captured by numerically computing their partial derivatives at 65 points scattered in the 4-dimensional parameter space {*a*, *s*, α, *P*} (Tables S1, S2, S3, S4 for β = 1; Tables S5 to S8 for β = 4/3).

When assuming *k* = 2/3 and β = 1, the qualitative impacts of *a*, *s*, and *P* on optimal solutions are, as far as we examined, identical with the patterns reported in the last section (Table 3). The most critical distinction between the present and *k* = β cases is that the definite sign of ∂*C*(*T*)*/∂α does change depending on the values of the other acidification-sensitive parameters (i.e., *a*, *s*, and *P*); it turns from negative to positive as acidification-driven costs increase (Fig. S1). Three additional differences from the case with *k* = β and the present case were also noted. First, the optimal proportion coefficient, δ*, becomes sensitivity to, and negatively depends on the defensible mortality risk, *P* (Table 3). Second, and contrary to the *k* = β case, the control variable, *u*, depends on the ontogenetic time, *t*, and thus increases with coccosphere size, *V*(*t*). As shown in Table 3, the optimal *u*-value at binary fission increases with increasing acidification-driven costs. Finally, the environmental sensitivity of *L*(T*) is also different from the *k* = β case; the probability of survival until binary fission decreases with increasing acidification-driven costs in this case.

**Table 3:** Optimal phenotypic responses to ocean acidification (*k* = 2/3, β = 1).

Acidification-sensitive parameters	T*	δ*	*u*(*T*)*	*V*(*T*)*	*C*(*T*)*	*L*(*T*)*	r*
Net production coefficient (*a*)	↓	↑	↓	↓	↓	↑	↑
Cost of calcification (*s*)	↑	↓	↑	↑	↑	↓	↓
Dissolution factor (α)	↑	↓	↑	↑	↓↑	↓	↓
Defensible mortality risk (*P*)	↑	↓	↑	↑	↑	↓	↓

Responses of optimal life history to the increase in acidification-sensitive parameters when *k* = 2/3, β = 1, *g*(*t*) = *P*/*C*(*t*)*^q^*, and *q* = 2 (1−*k*). Note that shown is a summary from Tables S1, S2, S3, and S4, and may not represent the global behavior due to lack of analytical tractability. See Table 2 for abbreviations.

In the case where *k* = 2/3 and β = 4/3, T*, δ*, *u*(*T*)*, *L*(T*), and r* show the exactly same qualitative dependencies on acidification-sensitive parameters as in the case with *k* = 2/3 and β = 1 (Table 4). As seen in Fig. 5, optimal coccolith size at binary fission, *C*(*T*)*, negatively depends on dissolution factor, α. A noteworthy outcome in this case is the parameter dependency of *V*(*T*)*. The optimal coccosphere size at binary fission has a convex function of α (see Fig. S2), in contrast to the two cases above, in which it always monotonically increases with increasing acidification-driven costs.

**Table 4:** Optimal phenotypic responses to ocean acidification (*k* = 2/3, β = 4/3).

Acidification-sensitive parameters	T*	δ*	*u*(*T*)*	*V*(*T*)*	*C*(*T*)*	*L*(*T*)*	r*
Net production coefficient (*a*)	↓	↑	↓	↓	↓	↑	↑
Cost of calcification (*s*)	↑	↓	↑	↑	↑	↓	↓
Dissolution factor (α)	↑	↓	↑	↓↑	↓	↓	↓
Defensible mortality risk (*P*)	↑	↓	↑	↑	↑	↓	↓

Responses of optimal life history to the increase in acidification-sensitive parameters when *k* = 2/3, β = 4/3, *g*(*t*) = *P*/*C*(*t*)*^q^*, and *q* = 2 (1−*k*). Note that shown is a summary from Tables S5, S6, S7, and S8, and may not represent the global behavior due to lack of analytical tractability. See Table 2 for abbreviations.

### The C/P ratio

When *k* = β (or α = 0) the calcification cost (*s*) is the only acidification-sensitive parameter that affects the C/P ratio ( = u*/*s*). It does so because the fractional energy allocation to coccolith production (u*) is independent of any acidification-sensitive parameters in this case (see Table 2). The inverse relationship between the C/P ratio and calcification cost holds in the cases where *k*≠β, as seen in equation (8). Additionally when *k*≠β, the C/P ratio becomes sensitive to acidification-sensitive parameters indirectly, because they affect the optimal energy allocation rate (u*). For example, u* increases with increasing *s*, and thus serves to increase the C/P ratio (see Table 4). The other acidification-sensitive parameters also affect the C/P ratio via the optimal allocation rate (see Fig. S3). Therefore, one cannot completely rule out the possibility that the C/P ratio may increase as ocean acidification progresses (Fig. S3). However, these effects acting through the energy allocation rate are limited in their magnitude, because u* can vary only within a small range of 0<*u*<1.

### Impacts on population-wise carbon fixation ability

**Figure 6:**
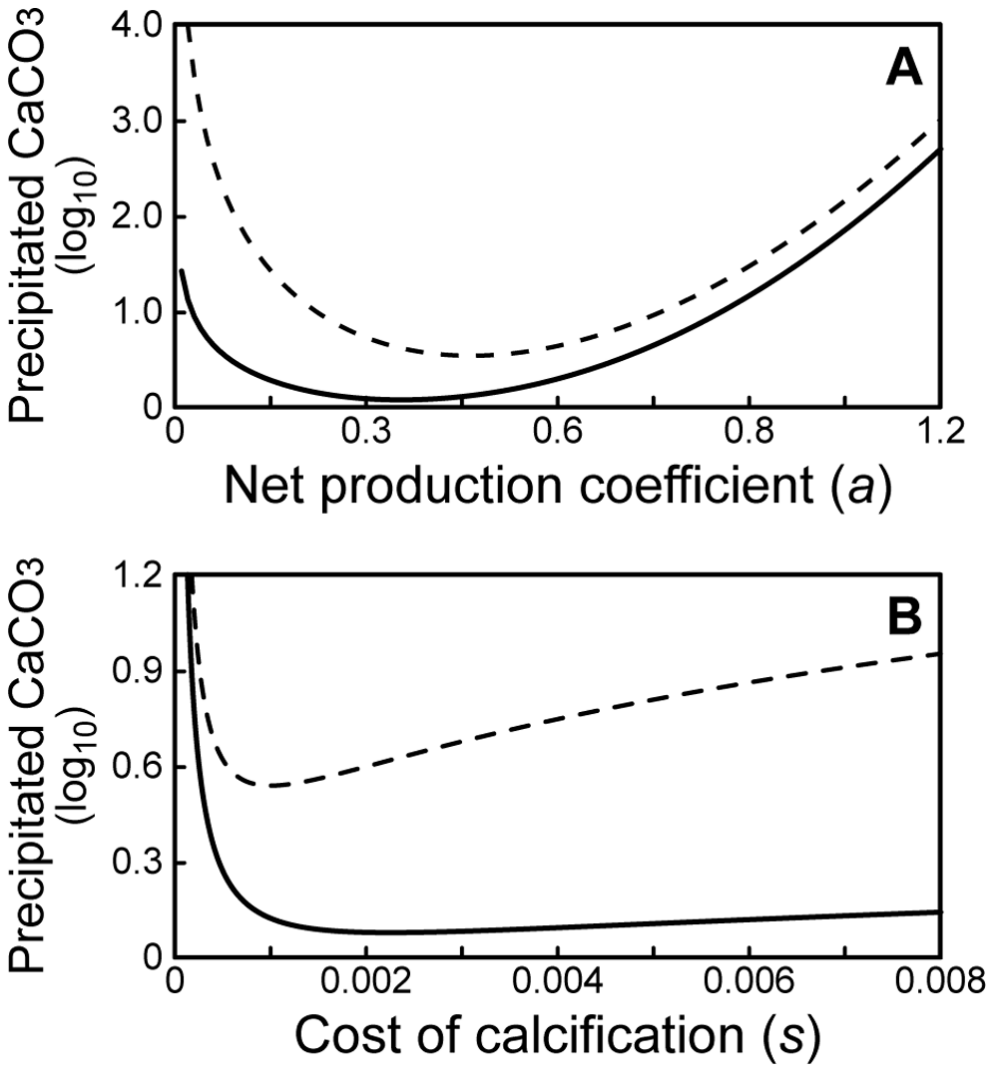
Environmental dependence of the total CaCO_3_ precipitated during a bloom of evolutionarily optimized coccolithophores. The population-wise carbon fixation (*W*) is given as the sum of the CaCO_3_ left during the bloom (*W*_1_) and the CaCO_3_ carried over until the end of the bloom (*W*_2_). (A) Both *W*_1_ (solid line) and *W*_2_ (dashed line) decrease and then increase with increasing net production coefficient (*a*) with *s* = 0.001. (B) Both *W*_1_ (solid line) and *W*_2_ (dashed line) depend on calcification cost (*s*) with L-shaped convex curves with *a* = 1.0. Common parameters: *k* = β = 2/3, *q* = 2 (1−*k*), α = 0, *P* = 1.0, *N*_0_ = 1.0, τ = 1.0. These figures assume the situation in which seawater remains oversaturated in CaCO_3_ (i.e., α = 0). Parameter dependencies in CaCO_3_-undersaturated seawater (i.e., α>0) are given in Figures S4 and S5.

The total amount of CaCO_3_ precipitated during the bloom (*W*) is given as the sum of coccoliths produced by the individuals died during the bloom (*W*_1_) and coccoliths held by the individuals surviving until the end of the bloom (*W*_2_). Unfortunately, these quantities are hardly tractable analytically, and require numerical computation to analyze their parameter dependences (Appendix S6), even assuming *k* = β and *q* = 2 (1−*k*). Assuming that *q* = 2 (1−*k*), both *W*_1_ and *W*_2_ first decrease and then increase with increasing acidification-driven costs, regardless of whether seawater is oversaturated in CaCO_3_ (Fig. 6) or not (Figs. S4 and S5). These patterns arise by the balancing effect between the acidification-driven increase in individual calcification and the decrease in the number of cells emerging during the bloom, accompanied by a longer generation time. One exception to these patterns is when β≫1: the increase in dissolution factor (α) causes monotonical decreases in both *W*_1_ and *W*_2_ (Fig. S5C) because a higher dissolution rate no longer causes the optimal coccolith size to be enlarged (see Table 4). The quantitative relationship between environmental variables and *W* is also sensitive to the blooming duration, τ: a longer τ always expands the parameter range in which the total amount of fixed CaCO_3_ decreases with increasing acidification-driven costs (see Figs. S4 and S5). This occurs because prolonged blooming duration intensifies the effect of population shrinkage caused by acidification, but does not affect an individual's calcification ability.

## Discussion

The most important finding from our calculation is that natural selection favors having more heavily calcified exoskeleton in response to increased acidification-driven costs in bloom-forming coccolithophores. In other words, as long as seawater is oversaturated in CaCO_3_, having a thicker coccolith layer is adaptive if the harmful effects due to higher calcification cost (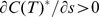), faster exoskeletal dissolution (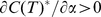), and/or increased defensible mortality risk (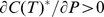) outweigh the beneficial effect by accelerated photosynthesis (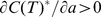). This pattern arises because, under higher acidification-driven costs, the fitness profit caused by lower mortality due to heavier coccoliths overcompensates the fitness loss resulting from a slower reproductive turnover associated with a longer generation time. Our theoretical conclusion appears counter-intuitive because most authors to date have postulated the working hypothesis that ocean acidification reduces net calcification rates in marine organisms [3]. Such a classical view seems to correctly predict a non-adaptive, plastic response commonly found in various taxa, but the scientific argument over evolutionary responses to ocean acidification is still at a starting point [29], [53]. Our calculation suggests that calcifying organisms can display the patterns opposite to non-adaptive, passive phenotypic responses, once evolutionary dynamics are considered.

Another noteworthy finding is that, depending on parameter values, acidification-driven phenotypic evolution does not necessarily decrease the total precipitated CaCO_3_ ( = *W*), suggesting that ocean acidification could possibly accelerates the alkalinity flux from the surface to deep water. The positive impact on the inorganic carbon pump is simply caused by the individual-based increase in calcification rate (i.e., increase in *C*(*T*)*). Our calculation also indicated that this positive effect may disappear if blooming duration (τ) gets longer as ocean acidification progresses. This makes it more difficult to predict the future carbon flux driven by coccolithophores, because the environmental factors that sustains a bloom (e.g., light; see [54]) could also be affected by the ongoing climate change.

Although the evolutionary response to ocean acidification found here is to increase the calcification rate of coccolithophores, this does not necessarily mean that there will be an increase in the ratio between calcification and photosynthesis (C/P ratio). A greater C/P ratio implies a greater likelihood that blooms serve as CO_2_ sources, i.e. calcification reaction generates CO_2_ in excess of its absorption by photosynthesis. Some studies based on numerical simulations have pointed out that the net CO_2_ uptake by coccolithophores will increase simply because the amount of calcification should be reduced by ocean acidification [55], [56]. This effect appears in our model as an increase in the calcification cost (*s*), which reduces the calcification rate. Focusing on the analytical case (*k* = β), the C/P ratio decreases with increasing calcification cost, but is independent of the other acidification-driven environmental changes (i.e., change in *a*, α, or *P*). Even in the cases with *k*≠β, the impact of calcification cost (*s*) on the C/P ratio is the most important, and is likely to dominate over the effects of the other acidification-sensitive parameters, which alter the C/P ratio indirectly by acting on the energy allocation rate (*u*) (see Results). In summary, our calculation suggests that the evolutionary response to ocean acidification is unlikely to enhance CO_2_ release during blooms, but leaves room for re-examination based on numerical considerations.

Our findings additionally provide a new insight into the interpretation of previously proposed empirical data. Iglesias-Rodriguez and colleagues presented the down-core data, in which average coccolith mass increases with rising atmospheric *p*CO_2_ after the Industrial Revolution [27], [57]. Grelaud et al. [58] have also demonstrated that increasing coccolith mass in six coccolithophorid species after 1917. As suggested by the authors, their observation should be understood by considering the evolutionary dynamics and our theoretical result is qualitatively consistent with this finding. The allopatric genotypic diversity within coccolithophorid species [59] may suggest that the observed long-term pattern reflects repeated invasions by close relatives with a better genotype [60]. Of course, the phenotypic change is not necessarily accompanied by genotypic replacement, because it can arise by a purely plastic response to the environmental change; it is possible that natural selection acts on reaction norms rather than phenotypic traits themselves. A recent study of seasonal variation in *E. huxleyi* morphology in the Aegean Sea again highlights that coccolithophores can have variable calcification levels dependent on the environmental conditions: There, either one population, with high acclamatory ability prevailed throughout the entire season or several different ecotypes were present, each of which favored specific conditions and so were dominant at different periods of the year [61].

Cautious consideration is required when using long term paleoceanographic data to evaluate the validity of our theory against past atmospheric CO_2_ concentration and nanoplankton fossil records. For example, Gibbs et al. [62] have rejected the hypothesis that less calcifying planktonic species are advantageous in terms of extinction and diversification during the Paleocene-Eocene Thermal Maximum (PETM), caused by a rapid increase in atmospheric CO_2_ concentration. Indeed, their conclusion is consistent with our result in the sense that natural selection does not favor a less calcifying strategy under ocean acidification conditions. However, the ocean acidification during the PETM was not accompanied by a decreased CaCO_3_ saturation state [63], and thus might be qualitatively different from the ongoing phenomenon. In addition, temperature increase at the PETM was several times higher than that for the past 200 years [27], [57], [58] and the thermal effect on coccolithophorid physiology cannot be ignored. Therefore, no compelling verification may be obtained by comparing the patterns in prehistoric geological records and the theoretical results drawn from our model that aims to evaluate the marginal effect of ocean acidification itself.

As with any theoretical study based on mathematical models, our conclusions largely depend on the assumptions that were made to assure biological plausibility and mathematical simplicity, some of which bear uncertainties due to the lack of empirical knowledge. For example, our model is based on a prevailing idea that coccoliths serve as a defensive organ [39], [64], but the exact form of mortality function in nature is still unknown. Also, we did not explicitly model an organic coating surrounding coccolith crystals, although this may serve to slow the rate of coccolith dissolution [47], [50]. Nevertheless, the observed positive relationship between acidification-driven costs and optimal coccolith size should be robust as far as the benefit of lower mortality (due to larger coccoliths) outweighs the cost of a longer generation time. Its robustness should be examined in future studies particularly against the assumptions that are biologically uncertain at this moment.

Our model does not explicitly parameterize a possible energetic advantage by the physiological coupling between photosynthesis and calcification, specifically reported in *Emiliania huxleyi* [50], [65]. This idea derives from the empirical finding that the proton produced by calcification (HCO_3_^−^+Ca_2_^+^→CaCO_3_+H^+^) is used to yield CO_2_, by reacting with HCO_3_^−^ in the medium via carbonic anhydrase (HCO_3_^−^+H^+^→CO_2_+H_2_O) to provide a substrate for photosynthesis. This route of CO_2_ supply is considered to be important, because *E. huxleyi* shows relatively low affinity for the CO_2_ dissolved in seawater [65]. The photosynthesis-calcification interaction can be incorporated into our model by replacing equation (1) with *dV*/*dt* = (1−*u*)*^z^aV^k^*, in which a lower *z* (0<*z*≤1) describes a larger energetic advantage. Since the optimal allocation rate (u*) is independent of, or little sensitive to, acidification (when α = 0 and when α>0, respectively), it is unlikely that this remodelling qualitatively alters the model's behavior, unless the parameter *z* itself is sensitive to acidification. In this case, *z* may gradually increase and approach 1 as ocean acidification progresses because the intracellular proton concentration should rise with decreasing seawater pH. Future studies should examine how the acidification-driven increase in *z* affects the optimal growth strategy of bloom-forming coccolithophores.

## Supporting Information

Appendix S1 Optimization.
(0.05 MB DOC)

Appendix S2 Optimal life history (*k* = β).
(0.22 MB DOC)

Appendix S3 Optimal life history (k≠β).
(0.03 MB DOC)

Appendix S4 Optimal life history (*k* = 2/3, β = 1).
(0.06 MB DOC)

Appendix S5 Optimal life history (*k* = 2/3, β = 4/3).
(0.06 MB DOC)

Appendix S6 Total precipitated CaCO_3_ (*q* = 2(1−*k*)).
(0.11 MB DOC)

Table S1 Qualitative dependencies of optimized parameters on the small increment of net production coefficient (*a*) at *k* = 2/3 and β = 1.
(0.03 MB PDF)

Table S2 Qualitative dependencies of optimized parameters on the small increment of calcification cost (*s*) at *k* = 2/3 and β = 1.
(0.03 MB PDF)

Table S3 Qualitative dependencies of optimized parameters on the small increment of dissolution coefficient (α) at *k* = 2/3 and β = 1.
(0.03 MB PDF)

Table S4 Qualitative dependencies of optimized parameters on the small increment of defensible mortality risk (*P*) at *k* = 2/3 and β = 1.
(0.03 MB PDF)

Table S5 Qualitative dependencies of optimized parameters on the small increment of net production coefficient (*a*) at *k* = 2/3 and β = 4/3.
(0.03 MB PDF)

Table S6 Qualitative dependencies of optimized parameters on the small increment of calcification cost (*s*) at *k* = 2/3 and β = 4/3.
(0.03 MB PDF)

Table S7 Qualitative dependencies of optimized parameters on the small increment of dissolution coefficient (α) at *k* = 2/3 and β = 4/3.
(0.03 MB PDF)

Table S8 Qualitative dependencies of optimized parameters on the small increment of defensible mortality risk (*P*) at *k* = 2/3 and β = 4/3.
(0.03 MB PDF)

Figure S1 Contour plots of optimal coccolith size at binary fission when *k* = 2/3 and β = 1.
(0.19 MB PDF)

Figure S2 Contour plots of optimal coccosphere size at binary fission when *k* = 2/3 and β = 4/3.
(0.21 MB PDF)

Figure S3 Marginal effects on the C/P ratio by (A) net production coefficient (*a*), (B) calcification rate (*s*), (C) dissolution coefficient (α), and (D) defensible mortality risk (*P*), respectively.
(0.15 MB PDF)

Figure S4 Behavior of *W*_1_ and *W*_2_ when *k* = 2/3 and β = 2/3.
(0.21 MB PDF)

Figure S5 Behavior of *W*_1_ and *W*_2_ when *k* = 2/3 and β = 4/3.
(0.16 MB PDF)
