# Supplementary material for: Increasing Costs Due to Ocean Acidification Drives Phytoplankton to Be More Heavily Calcified: Optimal Growth Strategy of Coccolithophores
Source: PLoS One. 2010 Oct 15;5(10):e13436. doi: 10.1371/journal.pone.0013436 (PMC2955539; doi:10.1371/journal.pone.0013436)
Supplement: Appendix S1 — Optimization. (0.05 MB DOC) [file pone.0013436.s001.doc]

**APPENDIX S1: Optimization**

In nature, coccolithophores in the coccolith-bearing phase split at non-zero intervals and their coccosphere and coccolith volumes have finite positive values during the life cycle. In other words, these variables have interior optima in the sense that the fitness, *r*, is maximized when *T*, *V*(*T*), and *C*(*T*) are in the open interval (ε, +∞) (an exception is *C*(*t*)* = 0 *t*). On the other hand, one may intuitively know that *r* is maximized when the generation time is asymptotically zero, if the mortality rate is size-independent, for example. Accordingly, we start the analysis by specifying the necessary conditions that the decision variables take interior optima.

The life history of coccolithophores is characterized by coccosphere and coccolith volumes at binary fission (*V*(*T*), *C*(*T*)), their ratio (δ), and generation time (*T*) in our model. Any two out of these four variables are functionally determined from the other two variables (see Appendix S2), which simplifies optimization. Using this fact, the intrinsic rate of increase given by equation (7) can be rewritten as a function of final volumes of coccosphere and coccolith, redefined as $\varphi\equiv V(T)$ and $\psi\equiv C(T)$:

$r=\frac{\ln2}{T(\varphi,\psi)}-\frac{1}{T(\varphi,\psi)}\int_{0}^{T(\varphi,\psi)} g(V(t,\varphi),C(t,\psi))dt$. [A1]

Regarding both $\varphi$ and $\psi$ as the arguments to be optimized, we take the partial derivatives of *r* with respect to these variables:

$$\frac{\partial r}{\partial\varphi}=-\frac{\left(\partial T/\partial\varphi\right)\ln2}{T(\varphi,\psi)^{2}}-\frac{\partial}{\partial\varphi}\left[\frac{1}{T(\varphi,\psi)}\int_{0}^{T(\varphi,\psi)} g(V(t,\varphi),C(t,\psi))dt\right]$$

$=-\frac{1}{T(\varphi,\psi)^{2}}\left[\frac{\partial T}{\partial\varphi}\ln2+\frac{\partial T}{\partial\varphi}\left\{T(\varphi,\psi)g(V(t,\varphi),C(t,\psi))-\int_{0}^{T(\varphi,\psi)} g(V(t,\varphi),C(t,\psi))dt\right\}\right.$ $\left.+T(\varphi,\psi)\int_{0}^{T(\varphi,\psi)} \frac{g(V(t,\varphi),C(t,\psi))}{V(t,\varphi)}\frac{V(t,\varphi)}{\varphi}dt\right]$. [A2]

Since $1/T(\varphi,\psi)^{2}\neq0$, $\partial r/\partial\varphi=0$ may be attained when the following equality holds:

$\frac{\partial T}{\partial\varphi}\left[\ln2+T(\varphi,\psi)g(\varphi,\psi)-\int_{0}^{T(\varphi,\psi)} g(V(t,\varphi),C(t,\psi))dt\right]+T(\varphi,\psi)\int_{0}^{T(\varphi,\psi)} \frac{\partial g(V(t,\varphi),C(t,\psi))}{\partial\varphi}dt=0$ [A3]

The counterpart for $\partial r/\partial\psi=0$ is:

$\frac{\partial T}{\partial\psi}\left[\ln2+T(\varphi,\psi)g(\varphi,\psi)-\int_{0}^{T(\varphi,\psi)} g(V(t,\varphi),C(t,\psi))dt\right]+T(\varphi,\psi)\int_{0}^{T(\varphi,\psi)} \frac{\partial g(V(t,\varphi),C(t,\psi))}{\partial\psi}dt=0$ [A4]

Analytical tractability of this system largely depends on whether the control variable (*u*) varies with ontogenetic time. Differentiating equation (3) with respect to *t* gives

$\frac{dC}{dt}=\delta\frac{dV}{dt}$. [A5]

Substituting the left and right sides of this equation with equations (1) and (2), respectively, and rearranging gives

$au/s=a\delta\left(1-u\right)+\alpha\delta^{\beta}V^{\beta-k}$, [A6]

which implies the control variable is time-invariant when *k* = β. In what follows, we begin with tackling the case where *k* = β (see Appendix S2), and then proceed to a general case assuming *k* ≠ β (see Appendix S3). Appendix S4 and Appendix S5 address special cases, in which β = 1 and β = 4/3, respectively, with *k* = 2/3 in both cases. Note that this conditional branching can be ignored only when α = 0.
