# Supplementary material for: Increasing Costs Due to Ocean Acidification Drives Phytoplankton to Be More Heavily Calcified: Optimal Growth Strategy of Coccolithophores
Source: PLoS One. 2010 Oct 15;5(10):e13436. doi: 10.1371/journal.pone.0013436 (PMC2955539; doi:10.1371/journal.pone.0013436)
Supplement: Appendix S2 — Optimal life history (k = β). (0.22 MB DOC) [file pone.0013436.s002.doc]

**APPENDIX S2: Optimal Life History (*k* = β)**

**1. Functional relationships between life history traits:** Life-history schedule is analytically tractable in this case. Equation [A6] can be solved for *u*:

$u=\frac{a\delta+\alpha\delta^{\beta}}{a\left(s^{-1}+\delta\right)}$, [B1-1]

and thus parameter values have to fulfill the following inequality by definition (i.e., *u* < 1):

$\delta<\left(a/s\alpha\right)^{1/\beta}$. [B1-2]

As far as *k* = β, the control variable, *u*, is time-invariant and differential equation (1) can be solved as:

$V(t)=\left[a\left(1-u\right)\left(1-k\right)t+V(0)^{1-k}\right]^{\frac{1}{1-k}}$, [B1-3]

after separating the variables *V* and *t* into both sides. From equation [B1-1], this can be rewritten as:

$V(t)=\left[\frac{\left(1-k\right)\left(as^{-1}-\alpha\delta^{\beta}\right)}{s^{-1}+\delta}t+\left(\frac{\varphi}{2}\right)^{1-k}\right]^{\frac{1}{1-k}}$, [B1-4]

where *V*(0) is substituted with $\varphi/2$. Substituting *t* with *T* in equation [B1-4], generation time, *T*, can be expressed as a function of the final coccosphere volume, $\varphi$, as:

$T=\frac{\left(1-2^{k-1}\right)\left(s^{-1}+\delta\right)}{\left(1-k\right)\left(as^{-1}-\alpha\delta^{\beta}\right)}\varphi^{1-k}$. [B1-5]

From equations [3] and [B1-4] we have:

$C(t)=\left[\frac{\left(1-k\right)\left(as^{-1}-\alpha\delta^{\beta}\right)}{\delta^{k}\left(\delta^{-1}s^{-1}+1\right)}t+\left(\frac{\psi}{2}\right)^{1-k}\right]^{\frac{1}{1-k}}=\left[Zt+\left(\psi/2\right)^{1-k}\right]^{\frac{1}{1-k}}$. [B1-6]

where

$Z\equiv\frac{\left(1-k\right)\left(as^{-1}-\alpha\delta^{\beta}\right)\delta^{1-k}}{s^{-1}+\delta}$. [B1-7]

Substituting *t* with *T* in equation [B1-6], and then solving for $\psi$ provides

$\psi=\left[\frac{ZT}{1-2^{k-1}}\right]^{\frac{1}{1-k}}$. [B1-8]

**2. Strategy of algebraic optimization:** As mentioned in the main text, the focal system has two arguments to be optimized, which can be arbitrarily chosen from $\varphi$, $\psi$, *T*, and δ. Simultaneous equations [A3] and [A4] potentially give the optimal solutions, but we below use only equation [A3] to assure algebraic tractability. Assuming that the mortality rate depends on coccolith size but not on coccosphere size (i.e., $\partial g(V(t,\varphi),C(t,\psi))/\partial C(t,\varphi)\neq0$) and $\partial g(V(t,\varphi),C(t,\psi))/\partial V(t,\varphi)=0$), the second term of the left-hand side in equation [A3] vanishes. Accordingly, equation [A3] holds when

$\ln2+T(\varphi,\psi)g(\varphi,\psi)-\int_{0}^{T(\varphi,\psi)} g(V(t,\varphi),C(t,\psi))dt=0$. [B2-1]

or

$\partial T/\partial\varphi=0$ [B2-2]

Interior optimal solutions should fulfill the function relationship constrained by either equation above, but another condition is required to complete optimization (instead of the reference to equation [A4]). This can be obtained by differentiating *r* with respect to *T* and equating it to zero, for example (see below).

**3. Consequence from equation (B2-2):** Differentiating equations [B1-5] with respect to $\varphi$, equation [B2-2] can be rewritten as:

$\frac{a-\alpha s\left(\psi/\varphi\right)^{\beta}-ak\left\{1+s\left(\psi/\varphi\right)\right\}}{\left(1-k\right)\varphi^{k}\left[a-\alpha s\left(\psi/\varphi\right)^{\beta}\right]^{2}\left(1-2^{k-1}\right)^{-1}}=0$, [B3-1]

of which the denominator of the left-hand side is non-zero because *k* ≠ 1. We thus obtain the following equation:

$\alpha\left(\psi/\varphi\right)^{\beta}+ak\left(\psi/\varphi\right)-a\left(1-k\right)/s=0$. [B3-2]

This equation has a closed-form solution for $\psi/\varphi$ (≡ δ) in special cases (e.g., *k* = β = 2/3), and makes equation [B1-7] simpler:

, [B3-3]

where δ* is the solution of equation [B3-2] with respect to $\psi/\varphi$ (≡ δ).

**4. Consequence from** $\partial r/\partial T=0$ **when *q* = 1 –** ***k*:** If we assign a particular function to the mortality, or *g*(*t*) = *P*/*C*(*t*)*^q^* and *q* = 1 – *k*, its definite integral from 0 to *T* is:

$L(T)=\exp\left[-\int_{0}^{T} \frac{P}{C(t)^{1-k}}dt\right]=\left[1+ZT\left(2/\psi\right)^{1-k}\right]^{-P/Z}$, [B4-1]

and the intrinsic rate of increase is:

$r=\frac{\ln2}{T}-\frac{P}{TZ}\ln\left[1+ZT\left(2/\psi\right)^{1-k}\right]=\frac{\left[Z-P\left(1-k\right)\right]\ln2}{\left(1-2^{k-1}\right)\psi^{1-k}}$. [B4-2]

Considering that the optimal proportion coefficient (δ*) has been determined from equation [B2-1] or [B2-2], the right-hand side in equation [B4-2] is maximized when $\psi$ is asymptotically zero, and thus coccosphere volume, $\varphi$, has no interior optimum, as well.

**5. Consequence from** $\partial r/\partial T=0$ **when *q* = 2 (1 –** ***k*):** Setting *q* = 2 (1 – *k*) instead of 1 – *k*, the probability of survival until binary fission is:

$$L(T)=\exp\left[-\int_{0}^{T} \frac{P}{C(t)^{2\left(1-k\right)}}dt\right]=\exp\left[-\frac{P}{\left(\psi/2\right)^{1-k}\left[Z+\left(\psi/2\right)^{1-k}T^{-1}\right]}\right]$$

$=\exp\left[-\frac{P\left(2^{1-k}-1\right)^{2}}{2^{1-k}Z^{2}T}\right]$. (from equation [B1-8]) [B5-1]

Consequently, the intrinsic rate of increase can be rewritten as:

$r=\frac{1}{T}\left[\ln2-\frac{P\left(2^{1-k}-1\right)^{2}}{2^{1-k}Z^{2}T}\right]$. [B5-2]

Equating $\partial r/\partial T$ to zero, and solving it for *T* provides the optimal relationship between generation time and proportion coefficient:

where $Z^{*}\equiv\left.Z\right|_{\delta=\delta^{*}}$. [B5-3]

We have the optimal coccolith volume from equations [B1-8] and [B5-3] as:

$\psi^{*}=\left[\frac{2P\left(2^{1-k}-1\right)}{Z^{*}\ln2}\right]^{\frac{1}{1-k}}$. [B5-4]

Substituting *T** in equation [B5-1] with the right-hand side of equation [B5-3], it turns out that the optimal probability of survival at binary fission is independent of any environmental factors:

$L(T^{*})=\exp\left[-\frac{\ln2}{2}\right]\approx0.707$. [B5-5]

This means that natural selection favors such a life history decision that coccolithophores split when the survival probability falls to a constant value.

Relationship between $\psi^{*}$ and $\varphi^{*}$ is given by substituting *ZT* in equation [B1-8] with *Z***T** derived from equations [B3-3] and [B5-3]:

$\psi^{*}=\left[\frac{2P\left(2^{1-k}-1\right)}{ak\left(1-k\right)\ln2}\right]^{\frac{1}{2\left(1-k\right)}}\sqrt{\varphi^{*}}$. [B5-6]

**6. Consequence from equation (C1) when *q* = 2 (1 –** ***k*):** Equation [B2-1] gives the optimal relationship between generation time and proportion coefficient:

. [B6-1]

Equating the right-hand side of equation [B5-3] to that of equation [B6-1] yields:

$2^{1-k}-2^{2-k}=1$, [B6-2]

which is never true if 0 < *k* < 1, and thus suggests that equation [B2-1] is not a necessary condition of optimal solutions (this was confirmed by numerical optimization; see below).

**7. Analytical consequences when *q* = 2 (1 –** ***k*) and α = 0:** In a special case where the dissolution factor is zero (i.e., α = 0), algebraic analysis progresses readily. In this case, the control variable given by equation [B1-1] is time-invariant, or *u* = 1/[(*s*δ)^-1^+1]. From equation [B3-2], we have the optimal proportion coefficient:

$\delta^{*}=\left(1-k\right)/ks$, [B7-1]

or equivalently,

$\psi^{*}=\frac{1-k}{ks}\varphi^{*}$, [B7-2]

suggesting that $\partial\delta^{*}/\partial a=0$, $\partial\delta^{*}/\partial s<0$, and $\partial\delta^{*}/\partial P=0$. Substituting $\psi^{*}$ in equation [B5-6] with the right-hand side of this equation, and solving it for $\varphi^{*}$ provides:

$\varphi^{*}=\left(\frac{ks}{1-k}\right)^{2}\left[\frac{2P\left(2^{1-k}-1\right)}{ak\left(1-k\right)\ln2}\right]^{\frac{1}{1-k}}$ [B7-3]

and thus

$\psi^{*}=\frac{ks}{1-k}\left[\frac{2P\left(2^{1-k}-1\right)}{ak\left(1-k\right)\ln2}\right]^{\frac{1}{1-k}}$. [B7-4]

Accordingly, we have $\partial\varphi^{*}/\partial a<0$, $\partial\varphi^{*}/\partial s>0$, $\partial\varphi^{*}/\partial P>0$, $\partial\psi^{*}/\partial a<0$, $\partial\psi^{*}/\partial s>0$, and $\partial\psi^{*}/\partial P>0$. The optimal generation time can be rewritten as

$T^{*}=\frac{2^{k}P}{\ln2}\left[\frac{2^{1-k}-1}{ak\left(1-k\right)}\left(\frac{ks}{1-k}\right)^{1-k}\right]^{2}$ [B7-5]

by substituting *Z** in equation [B5-3] with that in equation [B3-3]:

$Z^{*}=ak\left(1-k\right)\left(\frac{1-k}{ks}\right)^{1-k}$. [B7-6]

Equation [B7-5] provides $\partial T^{*}/\partial a<0$, $\partial T^{*}/\partial s>0$, and $\partial T^{*}/\partial P>0$.

**8. Analytical consequences when *q* = 2 (1 –** ***k*) and α ≥ 0:** We then consider general cases where the dissolution factor can be non-zero (α ≥ 0). Replacing $\psi/\varphi$ in equation [B3-2] with δ(*a*) and then taking the partial derivatives of both sides with respect to *a*, we can find the parameter dependency of optimal proportion coefficient:

, [B8-1]

where

$\frac{\partial\delta^{*}}{\partial a}\left\{\begin{matrix} >0 & \text{if} & \delta^{*}<\left(1-k\right)/ks \\ =0 & \text{if} & \delta^{*}=\left(1-k\right)/ks \\ <0 & \text{if} & \delta^{*}>\left(1-k\right)/ks \end{matrix}\right.$. [B8-2]

Equation [B3-2] can be rewritten as $\alpha\delta^{\beta}+ak\left[\delta-\left(1-k\right)/ks\right]=0$. Since *ak* > 0, we have

$\delta-\frac{1-k}{ks}<0$ $\left(\alpha>0\right)$ [B8-3]

and thus $\partial\delta^{*}/\partial a<0$ from equation [B8-2]. On the other hand, the dependencies of δ* on *s* and α are simply derived as:

[B8-4]

and

, [B8-5]

respectively. Equation [B3-2] implies $\partial\delta^{*}/\partial P=0$. The parameter dependencies of optimal generation time and optimal coccolith volume are given by differentiating equations [B5-3] and [B5-4] with respect to the focal variable.

Since both $\partial T^{*}/\partial Z^{*}<0$ and $\partial\psi^{*}/\partial Z^{*}<0$ hold regardless of α is zero or not, their environmental dependencies are determined by the definite sign of partial derivative of *Z** given by equation [B3-3]. Its partial derivative with respect to δ* is:

. [B8-6]

The partial derivative of δ* with respect to the focal environmental parameter also determines the parameter dependency of optimal strategies, and is already shown in equations [B8-1], [B8-4], and [B8-5]. From equation [B5-3], the dependencies of *T** on *a*, *s*, and α are given as:

$\frac{\partial T^{*}}{\partial a}=\frac{\partial T^{*}}{\partial Z^{*}}\left(\frac{\partial Z^{*}}{\partial a}+\frac{\partial Z^{*}}{\partial\delta^{*}}\frac{\partial\delta^{*}}{\partial a}\right)<0$, [B8-7]

$\frac{\partial T^{*}}{\partial s}=\frac{\partial T^{*}}{\partial Z^{*}}\left(\frac{\partial Z^{*}}{\partial s}+\frac{\partial Z^{*}}{\partial\delta^{*}}\frac{\partial\delta^{*}}{\partial s}\right)>0$, [B8-8]

and

$\frac{\partial T^{*}}{\partial\alpha}=\frac{\partial T^{*}}{\partial Z^{*}}\left(\frac{\partial Z^{*}}{\partial\alpha}+\frac{\partial Z^{*}}{\partial\delta^{*}}\frac{\partial\delta^{*}}{\partial\alpha}\right)>0$. [B8-9]

The counterparts to $\psi^{*}$ are derived from equation [B5-4]:

$\frac{\partial\psi^{*}}{\partial a}=\frac{\partial\psi^{*}}{\partial Z^{*}}\left(\frac{\partial Z^{*}}{\partial a}+\frac{\partial Z^{*}}{\partial\delta^{*}}\frac{\partial\delta^{*}}{\partial a}\right)<0$, [B8-10]

$\frac{\partial\psi^{*}}{\partial s}=\frac{\partial\psi^{*}}{\partial Z^{*}}\left(\frac{\partial Z^{*}}{\partial s}+\frac{\partial Z^{*}}{\partial\delta^{*}}\frac{\partial\delta^{*}}{\partial s}\right)>0$, [B8-11]

and

$\frac{\partial\psi^{*}}{\partial\alpha}=\frac{\partial\psi^{*}}{\partial Z^{*}}\left(\frac{\partial Z^{*}}{\partial\alpha}+\frac{\partial Z^{*}}{\partial\delta^{*}}\frac{\partial\delta^{*}}{\partial\alpha}\right)>0$. [B8-12]

Dependencies on *P* can be directly calculated from equations [B5-3] and [B5-4]:

$\frac{\partial T^{*}}{\partial P}=\frac{2^{k}\left(2^{1-k}-1\right)^{2}}{Z^{*}\ln2}>0$ and $\frac{\partial\psi^{*}}{\partial P}=\frac{\psi^{*}}{P\left(1-k\right)}>0$. [B8-13]

Since $\varphi^{*}=\psi^{*}/\delta^{*}$, it follows that

, [B8-14]

, [B8-15]

, [B8-16]

and

. [B8-17]

Substituting *L*(*T*) in equation [7] with *L*(*T**) in equation [B5-5], the intrinsic rate of population increase with optimal strategy can be written as:

$r^{*}=\frac{\ln2}{2T^{*}}$, [B8-18]

Letting *x* to be an element from a set of acidification-sensitive parameters, $\mathbf{x}:=\left\{a,s,\alpha,P\right\}$, equation [B8-18] suggests that $\mathrm{sgn}\left(\partial r^{*}/\partial x\right)=-\mathrm{sgn}\left(\partial T^{*}/\partial x\right)$. Accordingly, equations [B8-8], [B8-9], and [B8-13] suggest that the increases in acidification-driven costs lead to lower population growth rates.

Environmental dependency of the optimal energy allocation rate, *u**, can be analyzed by differentiating equation [B1-1] with respect to a focal acidification-sensitive parameter. Interestingly, *u** is independent of any acidification-sensitive parameters:

$\left.\frac{\partial u}{\partial a}\right|_{\delta=\delta^{*}}=\left.\frac{\partial u}{\partial s}\right|_{\delta=\delta^{*}}=\left.\frac{\partial u}{\partial\alpha}\right|_{\delta=\delta^{*}}=\left.\frac{\partial u}{\partial P}\right|_{\delta=\delta^{*}}=0$, [B8-19]

which is proved by referring to equations [B3-2], [B8-1], [B8-4], [B8-5].

**9. Numerical optimization when *q* = 2 (1 –** ***k*):** The analytical results above were confirmed by calculating optimal coccosphere and coccolith volumes from simultaneous equations [B3-2] and [B5-6] with *k* = β = 2/3. The behavior of these analytically obtained optima was then confirmed by numerical optimization, in which $\varphi$ and $\psi$ that maximize the intrinsic rate of population increase given by

$r=\frac{\left(1-k\right)\left[as^{-1}-\alpha\left(\psi/\varphi\right)^{\beta}\right]\ln2}{\varphi^{1-k}\left(1-2^{k-1}\right)\left[s^{-1}+\left(\psi/\varphi\right)\right]}-\frac{2^{1-k}P}{\psi^{2\left(1-k\right)}}$, [B9-1]

were examined on the platforms of R (ver. 2.8.1; for Windows, R Development Core Team) and Mathematica (ver. 7.0; for Windows, Wolfram Research). High resolution optima were determined using a stochastic iterated hill climbing program in Mathematica with their initial values roughly estimated by the built-in function, optim() in R.
