# Supplementary material for: Increasing Costs Due to Ocean Acidification Drives Phytoplankton to Be More Heavily Calcified: Optimal Growth Strategy of Coccolithophores
Source: PLoS One. 2010 Oct 15;5(10):e13436. doi: 10.1371/journal.pone.0013436 (PMC2955539; doi:10.1371/journal.pone.0013436)
Supplement: Appendix S3 — Optimal life history (k≠β). (0.03 MB DOC) [file pone.0013436.s003.doc]

**APPENDIX S3: Optimal Life History (*k* ≠ β)**

If we do not assume any relationship between *k* and β, multiple numerical techniques are required to compute the fitness. We here consider to calculate the intrinsic rate of population increase:

$r=\frac{1}{T}\left[\ln2-P\int_{0}^{T} C(t)^{-q}dt\right]$ [C1]

from a given set of $\left\{\varphi,\psi\right\}$. Solving equation [A6] for *u*, and substituting *V* with *C*/δ gives

$u\left(t\right)=\frac{\delta+\left(\alpha\delta^{k}/a\right)C(t)^{\beta-k}}{s^{-1}+\delta}$. [C2]

We have the growth equation of coccolith volume as

$\frac{dC}{dt}=\frac{a\delta^{1-k}C^{k}+\alpha C^{\beta}}{a+s\delta}-\alpha C^{\beta}$, [C3]

by substituting and *u* with equation [C2] and *V* with *C*/δ in equation (2). Accordingly, generation time, *T*, is given as the numerical solution at $C=\psi$ of the differential equation

$\frac{dt}{dC}=\left[\frac{a\delta^{1-k}C^{k}+\alpha C^{\beta}}{a+s\delta}-\alpha C^{\beta}\right]^{-1}$ [C4]

with the initial condition that $t(\psi/2)=0$, i.e.,

$T=t(\psi)$. [C5]

Equations [C3] and [C5] provide sufficient information for numerically solving the integral in equation [C1]. Optimal growth schedules at different sets of α and β were determined using a stochastic iterated hill climbing program on the platforms of Mathematica (ver. 7.0; for Windows, Wolfram Research).
