# Supplementary material for: Increasing Costs Due to Ocean Acidification Drives Phytoplankton to Be More Heavily Calcified: Optimal Growth Strategy of Coccolithophores
Source: PLoS One. 2010 Oct 15;5(10):e13436. doi: 10.1371/journal.pone.0013436 (PMC2955539; doi:10.1371/journal.pone.0013436)
Supplement: Appendix S4 — Optimal life history (k = 2/3, β = 1). (0.06 MB DOC) [file pone.0013436.s004.doc]

**APPENDIX S4: Optimal Life History (*k* = 2/3, β = 1)**

**1. Functional relationships between life history traits.** Once we relax the restriction that *k* = β, the algebraic analysis is hardly achieved except particular cases. An exception is the case that *k* = 2/3 and β = 1, in which the control variable continues to increase throughout the ontogeny:

$u\left(t\right)=\frac{\delta\left(a+\alpha V(t)^{1/3}\right)}{a\left(s^{-1}+\delta\right)}$, [D1-1]

as derived from equation [A6]. The counterpart to equation [B1-2] is:

$\delta V(t)^{1/3}<a/s\alpha$. ($0\leq t\leq T$) [D1-2]

Substituting *u* in differential equation (1) with the above equation, we obtain the ontogenetic trajectory of coccosphere volume:

$V\left(t\right)=\left[\frac{a}{s\alpha\delta}+\left\{\left(\frac{\varphi}{2}\right)^{1/3}-\frac{a}{s\alpha\delta}\right\}\exp\left(-\frac{\alpha\delta}{3\left(s^{-1}+\delta\right)}t\right)\right]^{3}$. [D1-3]

The counterpart to equation [B1-5] is:

$$T=\frac{3\left(s^{-1}+\delta\right)}{\alpha\delta}\ln\left[\left\{\left(\frac{\varphi}{2}\right)^{1/3}-\frac{a}{s\alpha\delta}\right\}/\left\{\varphi^{1/3}-\frac{a}{s\alpha\delta}\right\}\right]$$

$=\frac{1}{B}\ln\frac{\left(\varphi/2\right)^{1/3}-\left(A/\delta^{1/3}\right)}{\varphi^{1/3}-\left(A/\delta^{1/3}\right)}=\frac{1}{B}\ln\left[\frac{A-\left(\psi/2\right)^{1/3}}{A-\psi^{1/3}}\right]$ [D1-4]

and that to equation [B1-6] is:

$$C\left(t\right)=\left[\frac{a}{s\alpha\delta^{2/3}}+\left\{\left(\frac{\psi}{2}\right)^{1/3}-\frac{a}{s\alpha\delta^{2/3}}\right\}\exp\left(-\frac{\alpha\delta}{3\left(s^{-1}+\delta\right)}t\right)\right]^{3}$$

$=\left[A+\left\{\left(\psi/2\right)^{1/3}-A\right\}e^{-Bt}\right]^{3}$ [D1-5]

where

$A\equiv\frac{a}{s\alpha\delta^{2/3}}$ and $B\equiv\frac{s\alpha\delta}{3\left(1+s\delta\right)}$. [D1-6]

**2. Intrinsic rate of population increase when *q* = 1 –** ***k*:** Setting the mortality rate as *g*(*t*) = *P*/*C*(*t*)^1–^*^k^*, its definite integral from 0 to *T* is:

$$L(T)=\exp\left[-\int_{0}^{T} \frac{P}{C(t)^{1-k}}dt\right]=\exp\left[-\frac{P}{AB}\ln\left[\left(2/\psi\right)^{1/3}A\left(e^{BT}-1\right)+1\right]\right]$$

$=\left[\frac{A-\psi^{1/3}}{2^{1/3}A-\psi^{1/3}}\right]^{P/AB}$, (from equation [D1-4]) [D2-1]

and thus the intrinsic rate of increase is:

$r=B\ln2\left[\ln\left\{\frac{A-\left(\psi/2\right)^{1/3}}{A-\psi^{1/3}}\right\}\right]^{-1}\left(1-\frac{P}{3AB}\right)-\frac{P}{A}$. [D2-2]

Since the quantity in curly brackets increases with increasing $\psi$, *r* is maximized when $\psi$ is asymptotically zero, meaning that the life history strategy has no interior optimum.

**3. Intrinsic rate of population increase when *q* = 2 (1 –** ***k*):** If we assume that *q* = 2 (1 – *k*), the mortality rate is given as *g*(*t*) = *P*/*C*(*t*)^2/3^ and the probability of survival to binary fission, *L*(*T*), is:

$$L(T)=\exp\left[-\int_{0}^{T} \frac{P}{C(t)^{2\left(1-k\right)}}dt\right]=\exp\left[-\int_{0}^{T} P\left[A+\left\{\left(\psi/2\right)^{1/3}-A\right\}e^{-Bt}\right]^{-2}dt\right]$$

$=\exp\left[-\frac{P}{A^{2}B}\left[A\left(\frac{1}{\left(\psi/2\right)^{1/3}}-\frac{e^{BT}}{\left(\psi/2\right)^{1/3}+A\left(e^{BT}-1\right)}\right)+\ln\left(1+\frac{A\left(e^{BT}-1\right)}{\left(\psi/2\right)^{1/3}}\right)\right]\right]$ $=\exp\left[-\frac{P}{A^{2}B}\left[\frac{A\left(2^{1/3}-1\right)}{\psi^{1/3}}+\ln\left(\frac{2^{1/3}A-\psi^{1/3}}{A-\psi^{1/3}}\right)\right]\right]$. (from equation [D1-4]) [D3-1]

Accordingly, the fitness is expressed as:

$r=\left[B\ln2-\frac{P}{A^{2}}\left\{\frac{A\left(2^{1/3}-1\right)}{\psi^{1/3}}+\ln\left(\frac{2^{1/3}A-\psi^{1/3}}{A-\psi^{1/3}}\right)\right\}\right]/\ln\left[\frac{A-\left(\psi/2\right)^{1/3}}{A-\psi^{1/3}}\right]$. [D3-2]

Equation [D3-2] was used to grasp the model’s behavior by numerical optimization, in which the dependency of the optimal strategy on each environmental parameters was examined on the platforms of R (ver. 2.8.1; for Windows, R Development Core Team) and Mathematica (ver. 7.0; for Windows, Wolfram Research). Qualitative environmental dependencies of the optimal values were evaluated at the reference point (*a* = 0.1; *s* = 0.001; α = 0.0001; *P* = 1) and its surrounding 64 points set by doubling and halving one, two, or three acidification-sensitive parameters with respect to the values at the reference point (see Tables S1 to S4). The definite signs of partial derivatives at each point were determined by comparing them with optima at the two adjacent points determined by increasing and decreasing the corresponding coordinate value(s) by ten percent. At each point, high resolution optima for $\varphi$ and $\psi$ were determined using a stochastic iterated hill climbing program in Mathematica with their initial values roughly estimated by the built-in function, optim() in R. The summary of results is shown in Tables S1 to S4.
