# Supplementary material for: Increasing Costs Due to Ocean Acidification Drives Phytoplankton to Be More Heavily Calcified: Optimal Growth Strategy of Coccolithophores
Source: PLoS One. 2010 Oct 15;5(10):e13436. doi: 10.1371/journal.pone.0013436 (PMC2955539; doi:10.1371/journal.pone.0013436)
Supplement: Appendix S6 — Total precipitated CaCO3 (q = 2(1−k)). (0.11 MB DOC) [file pone.0013436.s006.doc]

**APPENDIX S6: Total Precipitated CaCO_3_ (*q* = 2 (1 –** ***k*))**

**1. Impact on the expected amount of CaCO_3_ left by a daughter cell that dies for a generation (Ξ) when *k* = β:** This quantity is expressed as:

$\Xi\equiv-\int_{0}^{T} C(t)\frac{dL}{dt}dt$ (from equation [4])

$=\int_{0}^{T} L(t)\frac{dC}{dt}dt-\left[C(t)L(t)\right]_{0}^{T}$ (partial derivative)

$=\int_{\psi/2}^{\psi} LdC-\psi\left(L(T)-\frac{1}{2}\right)$. (integration by substitution) [F1-1]

Considering that coccolithophores adopt the optimal strategy, the dependency of Ξ* on a given acidification-sensitive parameter, *x*, is given as:

$$\frac{\partial\Xi^{*}}{\partial x}=\int_{\psi^{*}/2}^{\psi^{*}} \left.\frac{\partial L(C)}{\partial x}\right|_{\begin{matrix} \psi=\psi^{*} \\ Z=Z^{*} \end{matrix}}dC+\frac{\partial\psi^{*}}{\partial x}\left[L(\psi^{*})-\frac{L(\psi^{*}/2)}{2}\right]-\frac{\partial\psi^{*}}{\partial x}\left(L(T^{*})-\frac{1}{2}\right)$$

$=\int_{\psi^{*}/2}^{\psi^{*}} \left.\frac{\partial L(C)}{\partial x}\right|_{\begin{matrix} \psi=\psi^{*} \\ Z=Z^{*} \end{matrix}}dC$. (because the second and third terms balance) [F1-2]

The probability of survival, *L*, as a function of *C* instead of *t* is given as:

$$L=\exp\left[-\int_{0}^{t} g(C(t'))dt'\right]$$

$=\exp\left[-\int_{0}^{t} \frac{P}{C(t')^{2\left(1-k\right)}}dt'\right]$ (from equation [6] with *q* = 2(1–*k*))

$=\exp\left[-\int_{\psi/2}^{C} \frac{P}{C'^{2\left(1-k\right)}}\left(\frac{dC'}{dt'}\right)^{-1}dC'\right]$ (integration by substitution)

$=\exp\left[-\frac{\left(1-k\right)P}{Z}\int_{\psi/2}^{C} C'^{k-2}dC'\right]$ (from equations [2])

$=\exp\left[-\frac{P}{Z}\left(\frac{1}{\left(\psi/2\right)^{1-k}}-\frac{1}{C^{1-k}}\right)\right]$. [F1-3]

The partial derivative of *L*(*C*) with respect to *a*, *s*, or α (denoted by *x*) is:

$\frac{\partial L(C)}{\partial x}=\frac{\partial Z}{\partial x}\frac{P}{Z^{2}}\left(\frac{1-k}{\left(\psi/2\right)^{1-k}}\frac{\partial\ln\psi/\partial x}{\partial\ln Z/\partial x}+\frac{1}{\left(\psi/2\right)^{1-k}}-\frac{1}{C^{1-k}}\right)\exp\left[-\frac{P}{Z}\left(\frac{1}{\left(\psi/2\right)^{1-k}}-\frac{1}{C^{1-k}}\right)\right]$. [F1-4]

Taking logarithm of equation [B5-4], and then differentiating with respect to *x* makes this equation simpler:

$\frac{\partial L(C)}{\partial x}=-\frac{\partial Z}{\partial x}\frac{P}{Z^{2}}\frac{1}{C^{1-k}}\exp\left[-\frac{P}{Z}\left(\frac{1}{\left(\psi/2\right)^{1-k}}-\frac{1}{C^{1-k}}\right)\right]$. [F1-5]

Thus it is evident that $\mathrm{sgn}\left(\partial L(C)/\partial x\right)=-\mathrm{sgn}\left(\partial Z/\partial x\right)$ on the closed interval $[\psi^{*}/2,\psi^{*}]$. On the other hand, the partial derivative of *L*(*C*) with respect to *P* is:

$\frac{\partial L(C)}{\partial P}=\frac{1}{Z}\left(\frac{P\left(1-k\right)}{\left(\psi/2\right)^{1-k}}\frac{\partial\ln\psi}{\partial P}-\frac{1}{\left(\psi/2\right)^{1-k}}+\frac{1}{C^{1-k}}\right)\exp\left[-\frac{P}{Z}\left(\frac{1}{\left(\psi/2\right)^{1-k}}-\frac{1}{C^{1-k}}\right)\right]$. [F1-6]

Taking logarithm of equation [B5-4], and then differentiating with respect to *P* suggests that $\partial\ln\psi^{*}/\partial P=\left[\left(1-k\right)P\right]^{-1}$, which simplifies equation [F1-6]:

$\frac{\partial L(C)}{\partial P}=\frac{1}{ZC^{1-k}}\exp\left[-\frac{P}{Z}\left(\frac{1}{\left(\psi/2\right)^{1-k}}-\frac{1}{C^{1-k}}\right)\right]$, [F1-7]

meaning that $\mathrm{sgn}\left(\partial L(C)/\partial P\right)>0$ on the closed interval $[\psi^{*}/2,\psi^{*}]$. From equations [F1-2], [F1-5] and [F1-7], we have $\partial\Xi^{*}/\partial a<0$, $\partial\Xi^{*}/\partial s>0$,$\partial\Xi^{*}/\partial\alpha>0$, and $\partial\Xi^{*}/\partial P>0$.

**2. Impact on the CaCO_3_ left during the bloom (*W*_1_) when *k* = β:** The partial derivative of *W*_1_* with respect to *x* is:

$\frac{\partial\ln W_{1}^{*}}{\partial x}=\frac{\partial\ln N_{\text{D}}^{*}}{\partial x}+\frac{\partial\ln\Xi^{*}}{\partial x}=-\frac{\tau}{T^{*}}\frac{\left(2L(T^{*})\right)^{\tau/T^{*}}\ln\left(2L(T^{*})\right)}{\left(2L(T^{*})-1\right)}\frac{\partial\ln T^{*}}{\partial x}+\frac{\partial\ln\Xi^{*}}{\partial x}$. [F2-1]

As analytically demonstrated above, $\mathrm{sgn}\left(\partial\ln T^{*}/\partial x\right)=-\mathrm{sgn}\left(\partial\ln\Xi^{*}/\partial\ln x\right)$ and thus no qualitative conclusion can be drawn for the definite sign of $\partial W_{1}^{*}/\partial x$, as far as *L*(*T**) > 0.5.

**3. Impact on the CaCO_3_ carried over until the end of the bloom (*W*_2_) when and *k* = β:** On the other hand, the partial derivative of ln *W*_2_* with respect to *x* is:

$\frac{\partial\ln W_{2}^{*}}{\partial x}=\frac{\partial\ln\psi^{*}}{\partial x}-\frac{\tau\ln\left[2L(T^{*})\right]}{T^{*}}\frac{\partial\ln T^{*}}{\partial x}$. [F3-1]

Taking logarithms of equations [B5-3] and [B5-4], respectively, and then differentiating with respect to *x* suggests that $\left(\partial\ln\psi^{*}/\partial x\right)/\left(\partial\ln T^{*}/\partial x\right)$= [2(1–*k*)]^–1^ if *x* is *a*, *s*, or α. It follows that

$\frac{\partial\ln W_{2}^{*}}{\partial x}\left\{\begin{matrix} >0 & \text{if} & \tau<T^{*}/2\left(1-k\right)\ln\left[2L(T^{*})\right] \\ =0 & \text{if} & \tau=T^{*}/2\left(1-k\right)\ln\left[2L(T^{*})\right] \\ <0 & \text{if} & \tau>T^{*}/2\left(1-k\right)\ln\left[2L(T^{*})\right] \end{matrix}\right.$, [F3-2]

in which 2 (1 – *k*) should be replaced with 1 – *k*, because $\left(\partial\ln\psi^{*}/\partial P\right)/\left(\partial\ln T^{*}/\partial P\right)$= (1 – *k*)^–1^. Focusing on *s*, α, and *P*, *W*_2_* has a convex upward function with a minimum point, at which the equality in [F3-2] is fulfilled, because $\partial T^{*}/\partial s>0$, $\partial T^{*}/\partial\alpha>0$, and $\partial T^{*}/\partial P>0$. The inequality signs in [F3-2] reverse when *x* = *a*, but $\partial\ln W_{2}^{*}/\partial a$ is convex upward against *a* because $\partial T^{*}/\partial a<0$ (see Fig. 6).

**4. Numerical evaluation of *W*_1_ and *W*_2_ when *k* = β:** In order to check the algebraic analysis above, the parameter dependencies of *W*_1_ and *W*_2_ were numerically examined by substituting equations (13) and (15) with numerically optimized $\psi$, *T*, and *L*(*T*) and assigning given *N*_0_ and τ. The computation of Ξ was not straightforward, and performed as follows. With *q* = 2 (1 – k), *L*(*t*) given by equation [B5-1] can be solved as:

$L(t)=\exp\left[-\frac{P}{\left(\psi/2\right)^{1-k}\left[Z+\left(\psi/2\right)^{1-k}t^{-1}\right]}\right]$. [F4-1]

Solving equation [B1-6] for *t*, and putting it into this equation provides

$L(C)=\exp\left[- \frac{P\left\{1-\left(\psi/2C\right)^{1-k}\right\}}{Z\left(\psi/2\right)^{1-k}}\right]$. [F4-2]

Substituting *L*(*C*) in the third equation of [F1-1] with the right-hand side of [F4-2], and numerically integrating the first term yields Ξ* by assigning optimal $\psi$ and *L*(*T*).

**5. Numerical evaluation of *W*_1_ and *W*_2_ when *k* = 2/3 and β = 4/3:** Once the equality between *k* and β breaks, little algebraic analysis can be achieved partly because *L*(*T**) is no longer independent of acidification-driven costs (see Tables 3 and 4). The computation of Ξ was performed in an analogous fashion to the last section; with *q* = 2 (1 – k), *L*(*t*) given by equation [E3-1] can be solved as:

$L(t)=\exp\left[-\frac{P}{A^{2}}\left(t+\frac{A^{2}-\left(\psi/2\right)^{2/3}}{B\left(\psi/2\right)^{1/3}\left[A+\left(\psi/2\right)^{1/3}\coth\left(Bt\right)\right]}\right)\right]$. [F5-1]

Solving equation [E1-3] for *t*, and putting it into this equation provides

$L(t)=\exp\left[-\frac{P}{A^{2}B}\left\{\tanh^{-1}\left(\frac{1-2^{-1/3}}{AC^{-1/3}-A^{-1}\left(\psi/2\right)^{1/3}}\right)+\frac{\left(2^{1/3}-1\right)\left(\left(2/\psi\right)^{1/3}A^{2}-\left(\psi/2\right)^{1/3}\right)}{A\left(2^{1/3}-1+\left(\psi/C\right)^{1/3}\right)-A^{-1}\left(\psi/2\right)^{2/3}}\right\}\right]$ [F5-2]

Substituting *L*(*C*) in the third equation of [F1-1] with the right-hand side of [F5-2], and numerically integrating the first term yields Ξ* by assigning optimal $\psi$ and *L*(*T*). *W*_1_ and *W*_2_ were computed by substituting equations (13) and (15) with numerically optimized $\psi$, *T*, and *L*(*T*) and assigning given *N*_0_ and τ.
