# Supplementary material for: Increasing Costs Due to Ocean Acidification Drives Phytoplankton to Be More Heavily Calcified: Optimal Growth Strategy of Coccolithophores
Source: PLoS One. 2010 Oct 15;5(10):e13436. doi: 10.1371/journal.pone.0013436 (PMC2955539; doi:10.1371/journal.pone.0013436)
Supplement: Table S1 — Qualitative dependencies of optimized parameters on the small increment of net production coefficient (a) at k = 2/3 and β = 1. (0.03 MB PDF) [file pone.0013436.s007.pdf]

Table S1. Qualitative dependencies of optimized parameters on the small increment of net production coefficient (a) at $k = 2/3$ and $\beta = 1$.

a	s	α	P	$\frac{\partial T^*}{\partial a}$	$\frac{\partial \delta^*}{\partial a}$	$\frac{\partial u(T^*)}{\partial a}$	$\frac{\partial \phi^*}{\partial a}$	$\frac{\partial \psi^*}{\partial a}$	$\frac{\partial L(T^*)}{\partial a}$	$\frac{\partial r*}{\partial a}$
0.10	0.0010	0.00010	1.0	↓	↑	↓	↓	↓	↑	↑
0.05	0.0010	0.00010	1.0	↓	↑	↓	↓	↓	↑	↑
0.10	0.0005	0.00010	1.0	↓	↑	↓	↓	↓	↑	↑
0.10	0.0010	0.00005	1.0	↓	↑	↓	↓	↓	↑	↑
0.10	0.0010	0.00010	0.5	↓	↑	↓	↓	↓	↑	↑
0.10	0.0010	0.00010	2.0	↓	↑	↓	↓	↓	↑	↑
0.10	0.0010	0.00020	1.0	↓	↑	↓	↓	↓	↑	↑
0.10	0.0020	0.00010	1.0	↓	↑	↓	↓	↓	↑	↑
0.20	0.0010	0.00010	1.0	↓	↑	↓	↓	↓	↑	↑
0.05	0.0005	0.00010	1.0	↓	↑	↓	↓	↓	↑	↑
0.05	0.0010	0.00005	1.0	↓	↑	↓	↓	↓	↑	↑
0.05	0.0010	0.00010	0.5	↓	↑	↓	↓	↓	↑	↑
0.05	0.0010	0.00010	2.0	↓	↑	↓	↓	↓	↑	↑
0.05	0.0010	0.00020	1.0	↓	↑	↓	↓	↓	↑	↑
0.05	0.0020	0.00010	1.0	↓	↑	↓	↓	↓	↑	↑
0.10	0.0005	0.00005	1.0	↓	↑	↓	↓	↓	↑	↑
0.10	0.0005	0.00010	0.5	↓	↑	↓	↓	↓	↑	↑
0.10	0.0005	0.00010	2.0	↓	↑	↓	↓	↓	↑	↑
0.10	0.0005	0.00020	1.0	↓	↑	↓	↓	↓	↑	↑
0.10	0.0010	0.00005	0.5	↓	↑	↓	↓	↓	↑	↑
0.10	0.0010	0.00005	2.0	↓	↑	↓	↓	↓	↑	↑
0.10	0.0010	0.00020	0.5	↓	↑	↓	↓	↓	↑	↑
0.10	0.0010	0.00020	2.0	↓	↑	↓	↓	↓	↑	↑
0.10	0.0020	0.00005	1.0	↓	↑	↓	↓	↓	↑	↑
0.10	0.0020	0.00010	0.5	↓	↑	↓	↓	↓	↑	↑
0.10	0.0020	0.00010	2.0	↓	↑	↓	↓	↓	↑	↑
0.10	0.0020	0.00020	1.0	↓	↑	↓	↓	↓	↑	↑
0.20	0.0005	0.00010	1.0	↓	↑	↓	↓	↓	↑	↑
0.20	0.0010	0.00005	1.0	↓	↑	↓	↓	↓	↑	↑
0.20	0.0010	0.00010	0.5	↓	↑	↓	↓	↓	↑	↑
0.20	0.0010	0.00010	2.0	↓	↑	↓	↓	↓	↑	↑
0.20	0.0010	0.00020	1.0	↓	↑	↓	↓	↓	↑	↑
0.20	0.0020	0.00010	1.0	↓	↑	↓	↓	↓	↑	↑
0.05	0.0005	0.00005	1.0	↓	↑	↓	↓	↓	↑	↑
0.05	0.0005	0.00010	0.5	↓	↑	↓	↓	↓	↑	↑
0.05	0.0005	0.00010	2.0	↓	↑	↓	↓	↓	↑	↑
0.05	0.0005	0.00020	1.0	↓	↑	↓	↓	↓	↑	↑
0.05	0.0010	0.00005	0.5	↓	↑	↓	↓	↓	↑	↑
0.05	0.0010	0.00005	2.0	↓	↑	↓	↓	↓	↑	↑
0.05	0.0010	0.00020	0.5	↓	↑	↓	↓	↓	↑	↑
0.05	0.0010	0.00020	2.0	↓	↑	↓	↓	↓	↑	↑
0.05	0.0020	0.00005	1.0	↓	↑	↓	↓	↓	↑	↑
0.05	0.0020	0.00010	0.5	↓	↑	↓	↓	↓	↑	↑
0.05	0.0020	0.00010	2.0	↓	↑	↓	↓	↓	↑	↑
0.05	0.0020	0.00020	1.0	↓	↑	↓	↓	↓	↑	↑
0.10	0.0005	0.00005	0.5	↓	↑	↓	↓	↓	↑	↑
0.10	0.0005	0.00005	2.0	↓	↑	↓	↓	↓	↑	↑
0.10	0.0005	0.00020	0.5	↓	↑	↓	↓	↓	↑	↑
0.10	0.0005	0.00020	2.0	↓	↑	↓	↓	↓	↑	↑
0.10	0.0020	0.00005	0.5	↓	↑	↓	↓	↓	↑	↑
0.10	0.0020	0.00005	2.0	↓	↑	↓	↓	↓	↑	↑
0.10	0.0020	0.00020	0.5	↓	↑	↓	↓	↓	↑	↑
0.10	0.0020	0.00020	2.0	↓	↑	↓	↓	↓	↑	↑
0.20	0.0005	0.00005	1.0	↓	↑	↓	↓	↓	↑	↑
0.20	0.0005	0.00010	0.5	↓	↑	↓	↓	↓	↑	↑
0.20	0.0005	0.00010	2.0	↓	↑	↓	↓	↓	↑	↑
0.20	0.0005	0.00020	1.0	↓	↑	↓	↓	↓	↑	↑
0.20	0.0010	0.00005	0.5	↓	↑	↓	↓	↓	↑	↑
0.20	0.0010	0.00005	2.0	↓	↑	↓	↓	↓	↑	↑
0.20	0.0010	0.00020	0.5	↓	↑	↓	↓	↓	↑	↑
0.20	0.0010	0.00020	2.0	↓	↑	↓	↓	↓	↑	↑
0.20	0.0020	0.00005	1.0	↓	↑	↓	↓	↓	↑	↑
0.20	0.0020	0.00010	0.5	↓	↑	↓	↓	↓	↑	↑
0.20	0.0020	0.00010	2.0	↓	↑	↓	↓	↓	↑	↑
0.20	0.0020	0.00020	1.0	↓	↑	↓	↓	↓	↑	↑
