# Supplementary material for: Increasing Costs Due to Ocean Acidification Drives Phytoplankton to Be More Heavily Calcified: Optimal Growth Strategy of Coccolithophores
Source: PLoS One. 2010 Oct 15;5(10):e13436. doi: 10.1371/journal.pone.0013436 (PMC2955539; doi:10.1371/journal.pone.0013436)
Supplement: Figure S2 — Contour plots of optimal coccosphere size at binary fission when k = 2/3 and β = 4/3. (0.21 MB PDF) [file pone.0013436.s016.pdf]

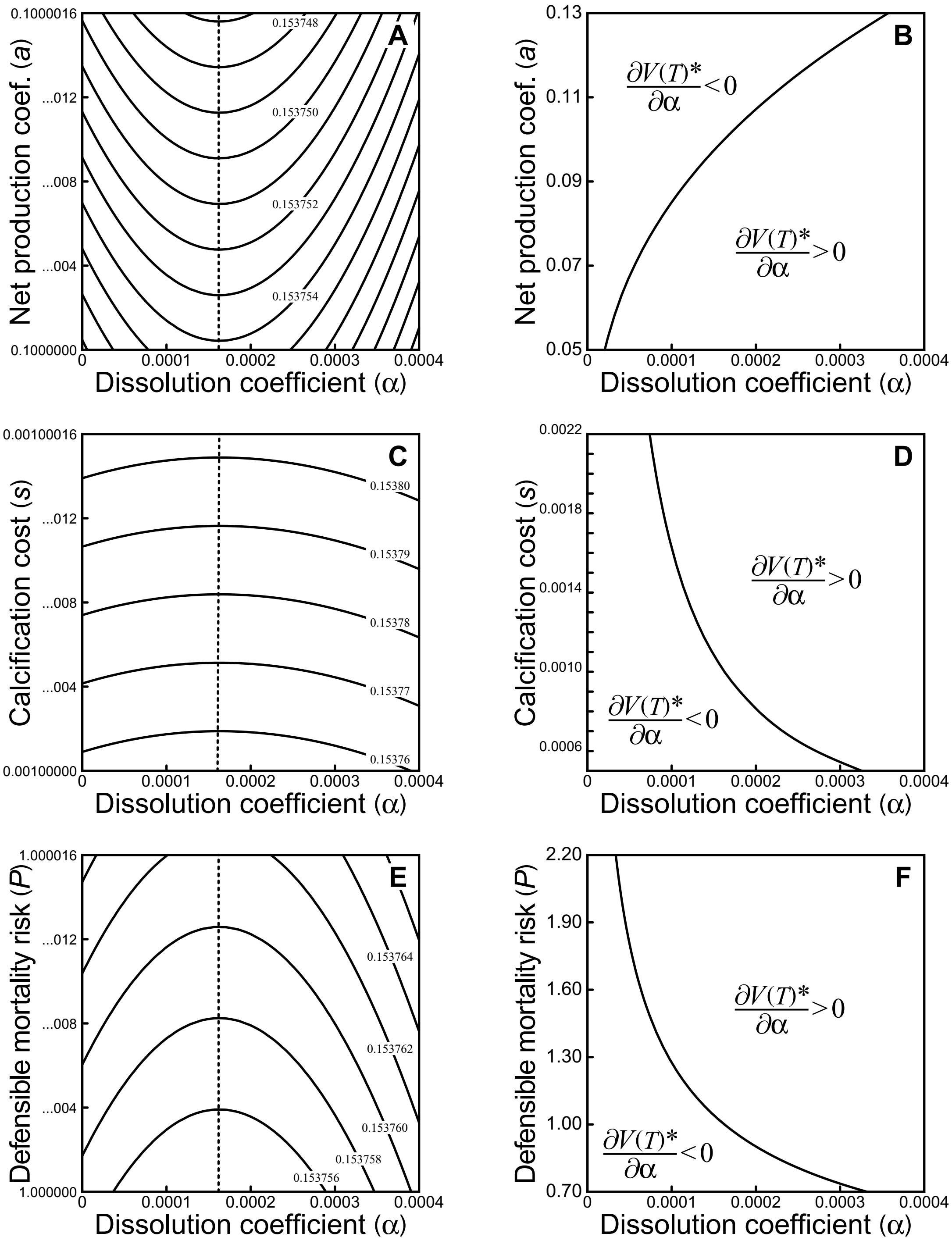

Figure S2. Contour plots of optimal coccosphere size at binary fission when $k = 2/3$ and $\beta = 4/3$. $V(T)^*$ is given as functions of dissolution coefficient, α, and (**A**) net production coefficient, a, (**C**) calcification cost, s, and (**E**) defensible mortality risk, P, respectively, in which $\partial V(T)^*/\partial\alpha = 0$ fulfills on the dotted lines. To emphasize its parameter dependencies, the dotted lines are redrawn as solid lines in (**B**), (**D**), and (**F**). Note that ordinate scales differ between the left and right panels. Common parameter values: $a = 0.1$, $s = 0.001$, $P = 1.0$, $k = 2/3$, $\beta = 4/3$, $q = 2/3$, unless designated in repsective panels.
