# Supplementary material for: Increasing Costs Due to Ocean Acidification Drives Phytoplankton to Be More Heavily Calcified: Optimal Growth Strategy of Coccolithophores
Source: PLoS One. 2010 Oct 15;5(10):e13436. doi: 10.1371/journal.pone.0013436 (PMC2955539; doi:10.1371/journal.pone.0013436)
Supplement: Figure S3 — Marginal effects on the C/P ratio by (A) net production coefficient (a), (B) calcification rate (s), (C) dissolution coefficient (α), and (D) defensible mortality risk (P), respectively. (0.15 MB PDF) [file pone.0013436.s017.pdf]

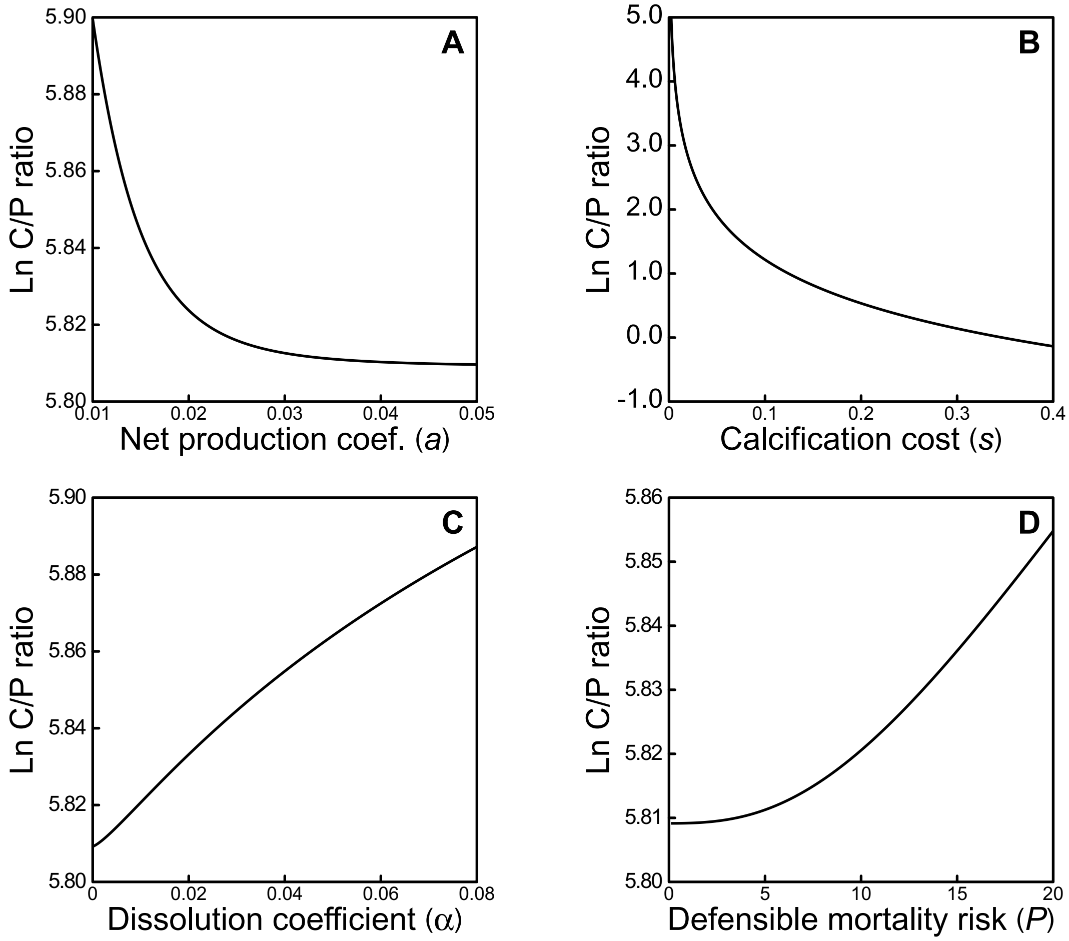

Figure S3. Marginal effects on the C/P ratio by (**A**) net production coefficient (a), (**B**) calcification rate (s), (**C**) dissolution coefficient (α), and (**D**) defensible mortality risk (P), respectively. The natural logarithm of the C/P ratio, given by equation (8), equals the sum of $\ln\left(\int_0^T u(t)V(t)^k L(t)dt / \int_0^T V(t)^k L(t)dt\right)$ and $-\ln s$. The environmental dependency of the C/P ratio is simply determined by that of u^* in panels (**A**), (**C**) and (**D**), in which s keeps constant. On the other hand, the C/P ratio decreases with increasing s, mainly reflecting the behavior of $-\ln s$ rather than $\ln\left(\int_0^T u(t)V(t)^k L(t)dt / \int_0^T V(t)^k L(t)dt\right)$ in panel (**B**). Common parameter values: $a = 1.0$, $s = 0.001$, $\alpha = 0.0001$, $P = 1.0$, $k = 2/3$, $\beta = 4/3$, $q = 2/3$, unless designated in repsective panels.
