# Supplementary material for: Increasing Costs Due to Ocean Acidification Drives Phytoplankton to Be More Heavily Calcified: Optimal Growth Strategy of Coccolithophores
Source: PLoS One. 2010 Oct 15;5(10):e13436. doi: 10.1371/journal.pone.0013436 (PMC2955539; doi:10.1371/journal.pone.0013436)
Supplement: Figure S5 — Behavior of W 1 and W 2 when k = 2/3 and β = 4/3. (0.16 MB PDF) [file pone.0013436.s019.pdf]

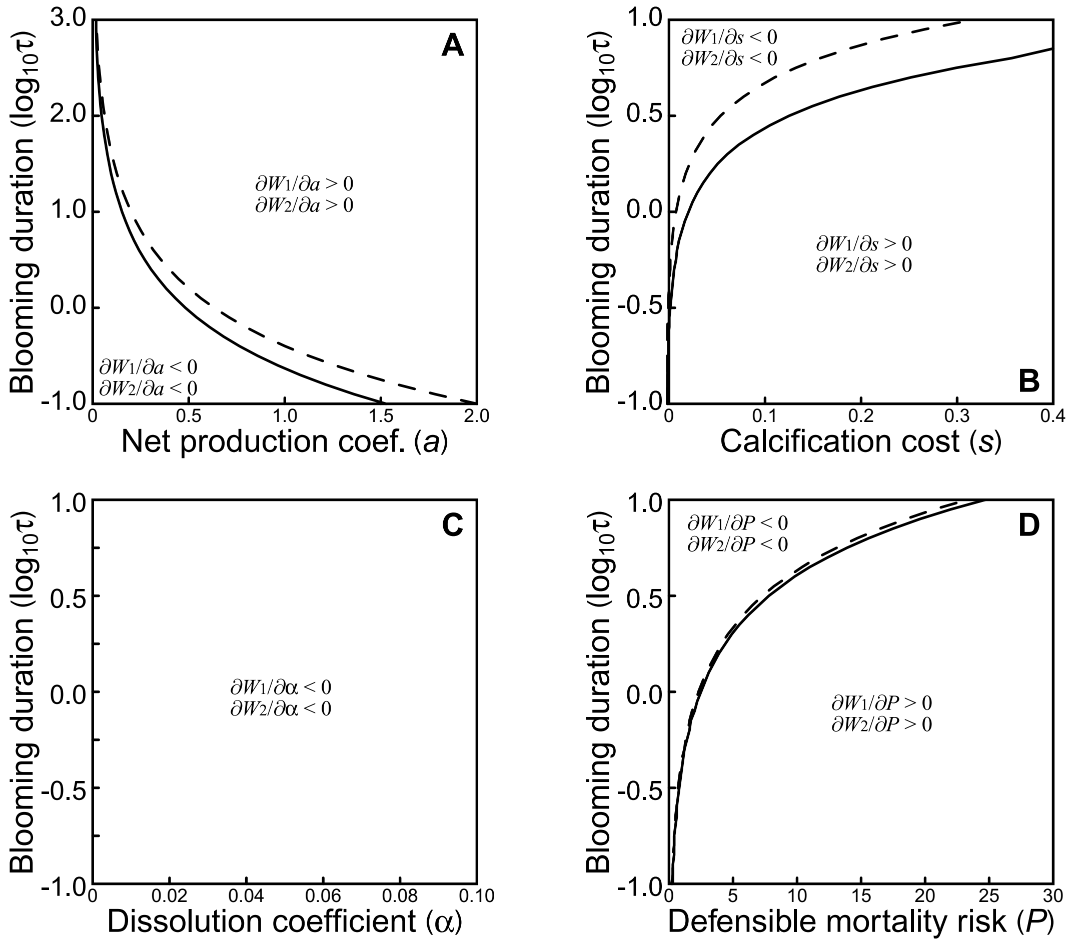


Figure S5. Behavior of $W_1$ and $W_2$ when $k = 2/3$ and $\beta = 4/3$. $W_1$ and $W_2$ are plotted against blooming duration ($\tau$) and (**A**) net production coefficient ($a$), (**B**) calcification rate ($s$), (**C**) dissolution coefficient ($\alpha$), and (**D**) defensible mortality risk ($P$), respectively. Partial derivative of $W_1$ with respect to the focal acidification-sensitive parameter is zero on solid lines. Similarly, partial derivative of $W_2$ is zero on dashed lines. Common parameter values: $a = 1.0$, $s = 0.001$, $\alpha = 0.0001$, $P = 1.0$, $k = 2/3$, $\beta = 4/3$, $q = 2/3$, $N_0 = 1.0$, unless designated in repsective panels.
